# Supplementary material for: Defects in the cell wall and its deposition caused by loss-of-function of three RLKs alter root hydrotropism in Arabidopsis thaliana
Source: Nat Commun. 2024 Mar 26;15:2648. doi: 10.1038/s41467-024-46889-2 (PMC10966064; doi:10.1038/s41467-024-46889-2)
Supplement: Supplementary file 1 — Supplementary Information [file 41467_2024_46889_MOESM1_ESM.docx]

**Supplementary Information for**

**Defects in the cell wall and its depositions caused by loss-of-function of three RLKs alter root hydrotropism in *Arabidopsis thaliana***

**Jinke Chang****^1, 3^, Xiaopeng Li^2^, Juan Shen^1^, Jun Hu^1^, Liangfan Wu^1^, Xueyao Zhang^1^, Jia Li^1, 2, 3 *^**

^1^ Ministry of Education Key Laboratory of Cell Activities and Stress Adaptations, School of Life Sciences, Lanzhou University, Lanzhou 730000, China.

^2^ Guangdong Provincial Key Laboratory of Plant Adaptation and Molecular Design, School of Life Sciences, Guangzhou University, Guangzhou 510006, China.

^3^ Gansu Key Laboratory of Gene Editing for Breeding, School of Life Sciences, Lanzhou University, Lanzhou 730000, China.

**^*^ Correspondence:** Jia Li

**Email:** [lijia@gzhu.edu.cn](mailto:lijia@gzhu.edu.cn)

**This file includes:**

Supplementary Figure 1 to Figure 28

Supplementary Table 1 to Table 3


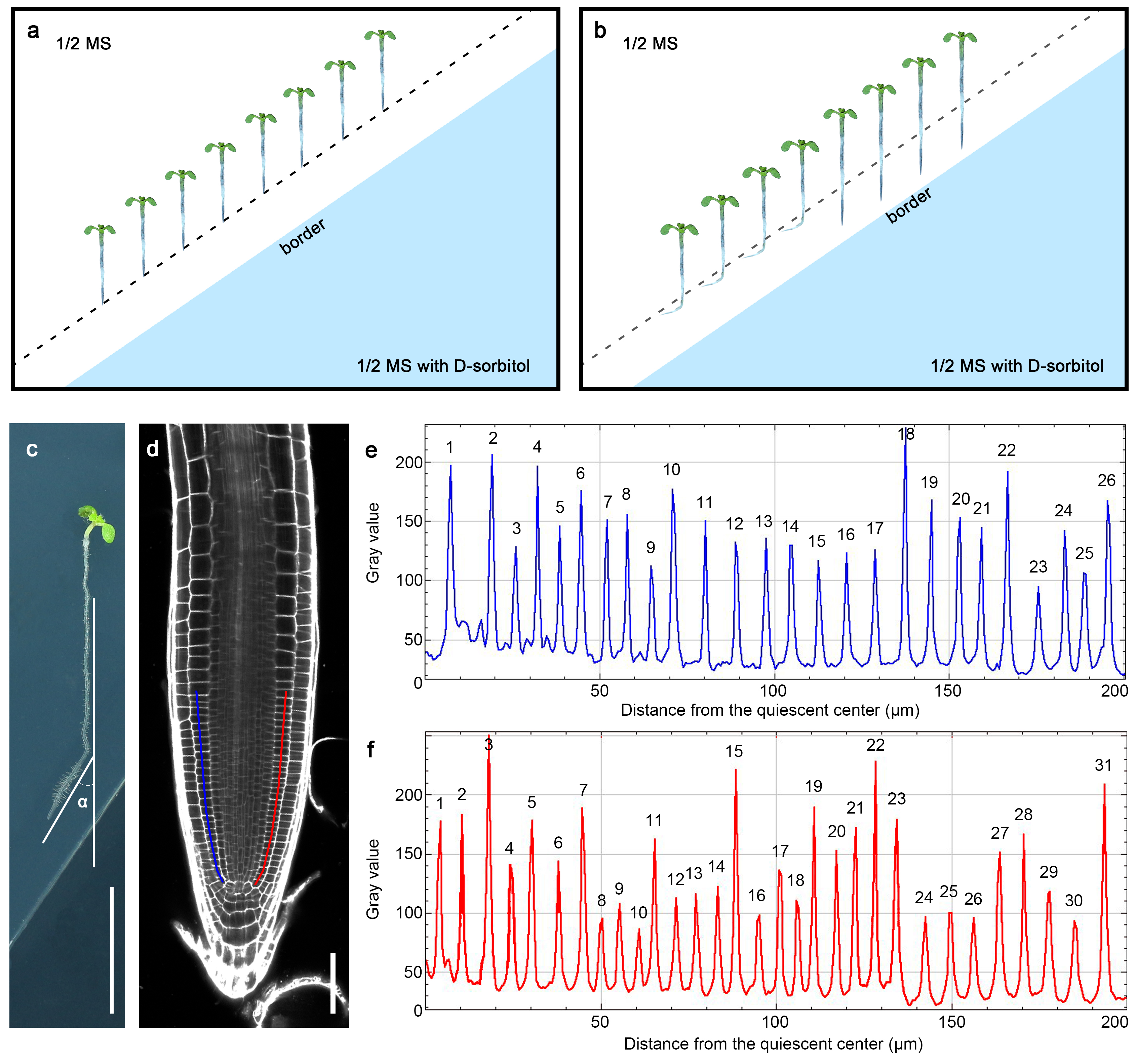


**Supplementary Figure 1 | Methods used for hydrostimulation treatment, root bending curvature measurement, and meristem cortex cell number counting.**

**a**-**c**, 1/2 MS medium supplemented with 1% sucrose and 1% agar (w/v) was pulled in a Petri dish. After solidified, right bottom side of the medium was removed and replaced with 1/2 MS medium supplemented with 1% sucrose, 1% agar (w/v), and D-sorbitol. Such a medium is named as hydrostimulating (moisture gradient) medium. To perform hydrostimulating analysis, four-day-old seedlings were transferred to the hydrostimulating medium with root tips 0.5 cm away from the border (a), seedlings were then vertically grown on the medium for specified time periods (b), photographed (c), and root growth curvatures were measured by ImageJ (c). The root tips of moisture gradient sensitive seedlings bend to high water potential side (four seedlings showed at the left bottom side in b), root tips of moisture gradient insensitive mutants grow straightly into the low water potential side (four seedlings showed at the top right side in b). **d**-**f**, cell numbers were counted by the peaks of cell wall fluorescence by using Image J software after propidium iodide (PI) staining. For example, a hydrostimulated root showing in (d), the convex side contains 31 cortex cells (f), and the concave side contains 26 cortex cells (e) within a 200 μm meristematic zone starting from the quiescent center. Scale bar represents 5 mm in (c). and 50 µm in (d).

**
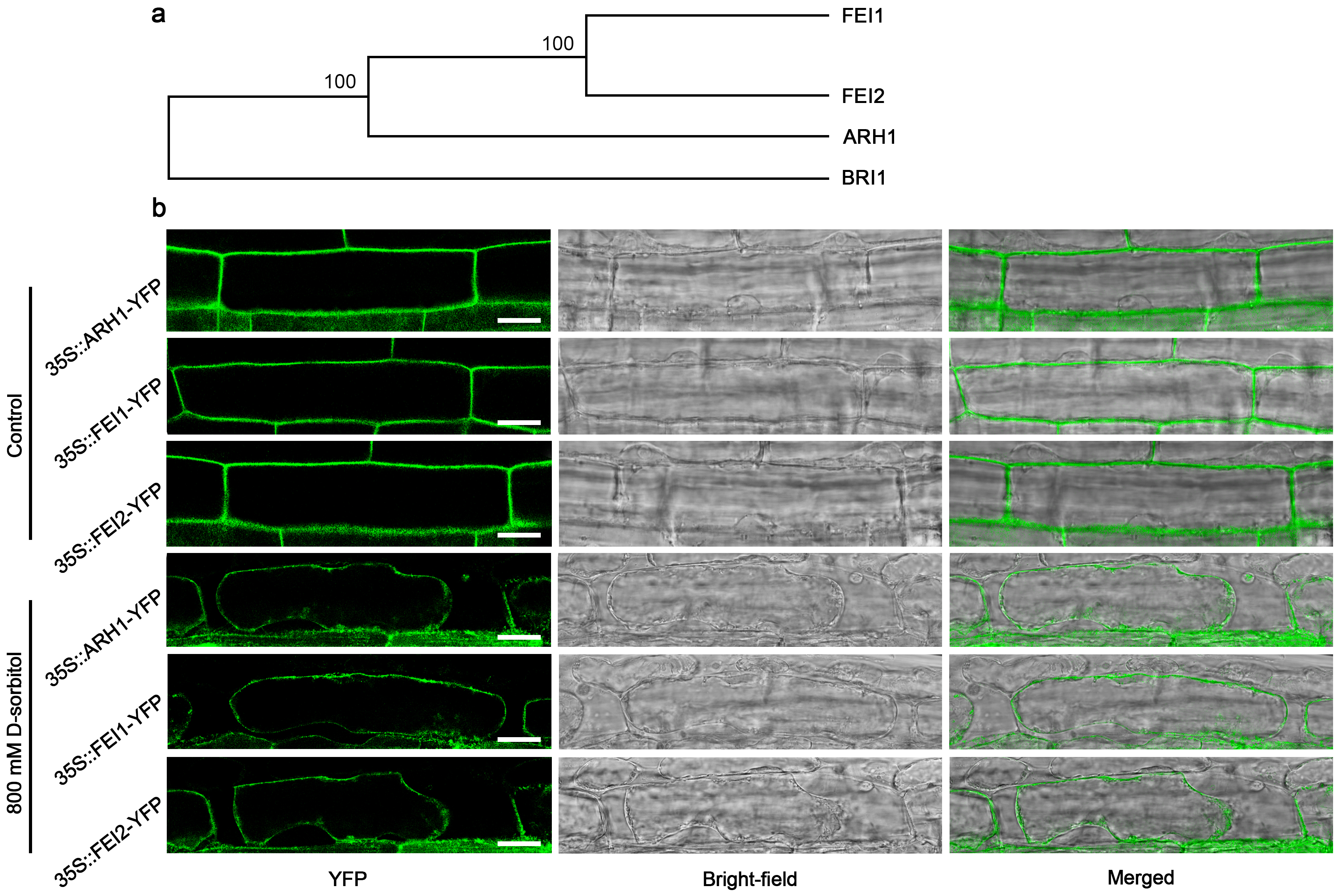
**

**Supplementary Figure 2 | ARH1, FEI1, and FEI2 are localized on the plasma membrane.**

**a**, A phylogenetic tree of ARH1, FEI1, and FEI2 was constructed using the MEGA7 software based on their full-length amino acid sequences. BRI1 was used as an outgroup control. **b**, The subcellular localizations of ARH1-YFP, FEI1-YFP, and FEI2-YFP were determined in the root elongation zones of transgenic plants overexpressing their corresponding coding genes. Plasma localization was determined by plasmolysis using 800 mM D-sorbitol. Three biological replicates were carried out. Scale bar represents 10 µm.


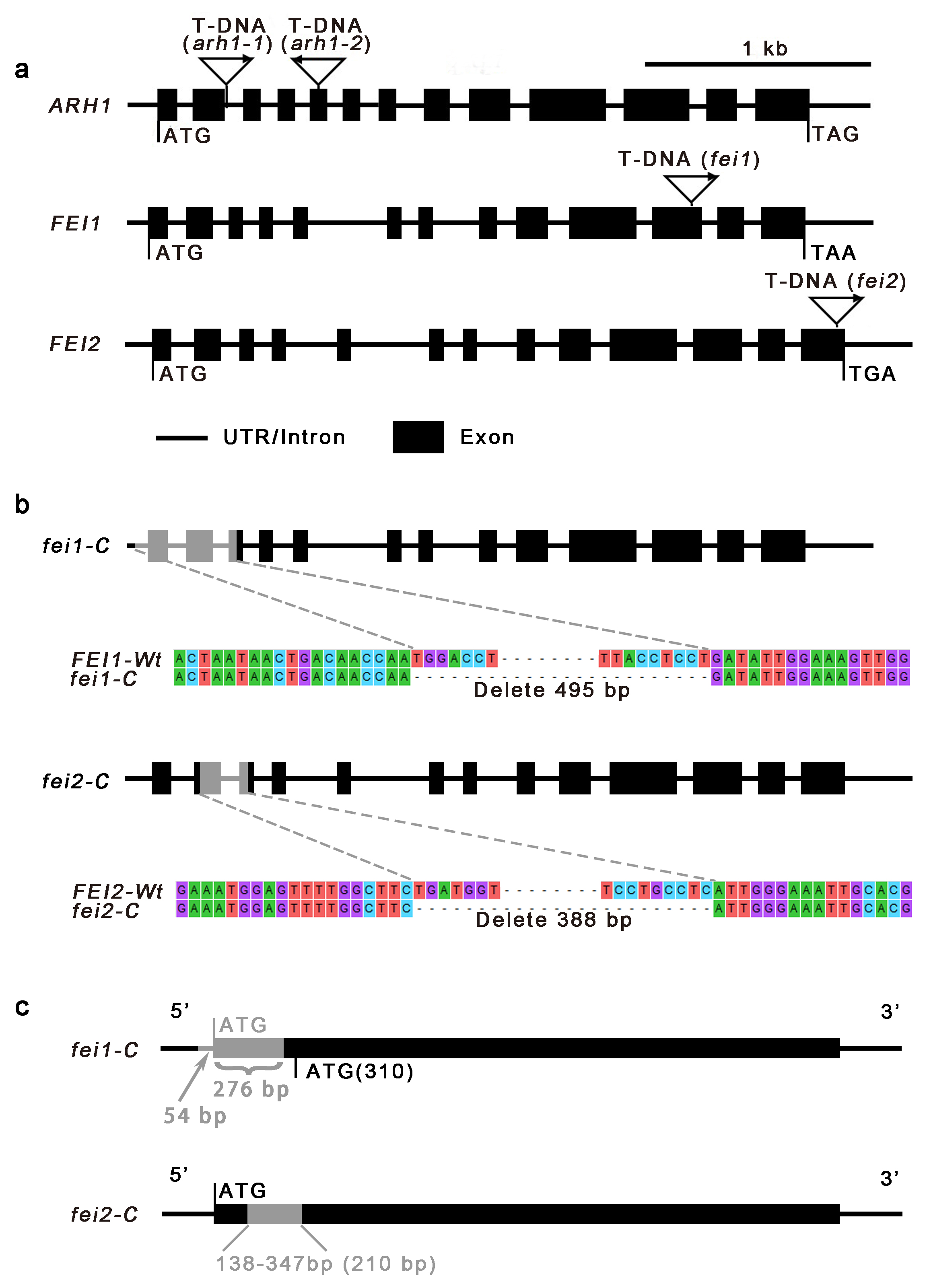


**Supplementary Figure 3 | The detailed mutation alleles of *ARH1*, *FEI1*, and *FEI2*.**

**a**, Diagrams showing the T-DNA insertion sites of mutant alleles of *ARH1*, *FEI1*, and *FEI2*. Boxes represent exons and triangles indicate the positions of T-DNA insertions and the T-DNA orientations are indicated with arrows. **b**, Gene editing details in the genomes of *fei1-C* and *fei2-C*, both of which were generated by a CRISPR-Cas9 system. The deleted sequences were marked in gray. **c**, Diagrams of cDNA sequence showing the gene editing details of *fei1-C* and *fei2-C*. In *fei1-C*, the deletion includes 54 bp of 5’-UTR and 276 bp of encoding sequence including the initiation codon ATG, the next ATG sequence is started from the 310^th^ nucleotide of the first ATG. In *fei2-C*, the 210 bp deleted sequence is from nucleotide 138^th^ to 347^th^ counting from the initiation codon ATG.


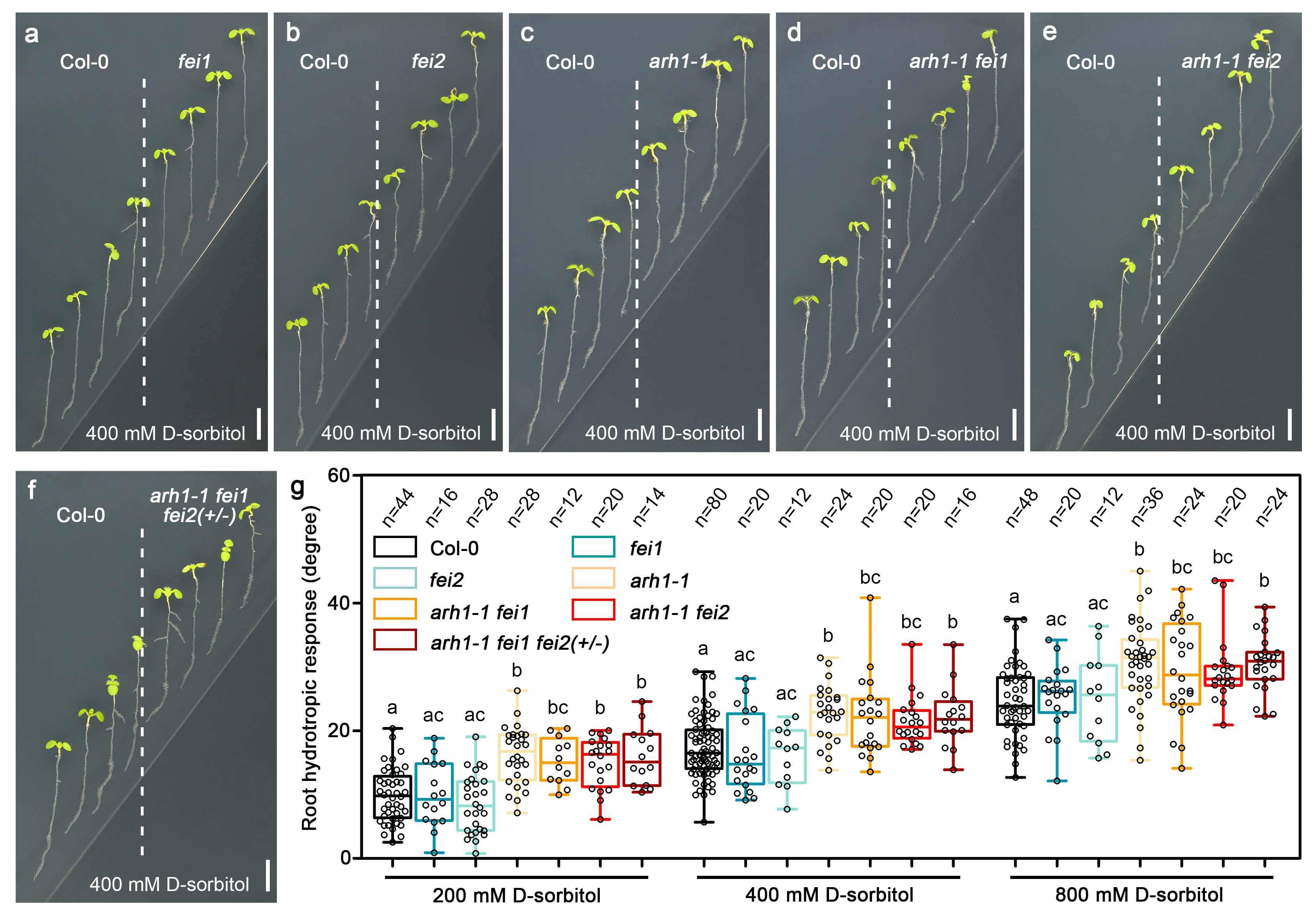


**Supplementary Figure 4 | Various single and double mutants of T-DNA insertion lines of *ARH1*, *FEI1*, and *FEI2* show enhanced response to hydrostimulation treatments.**

**a-f**, Hydrotropic response of representative wild-type (Col-0) and indicated mutants after hydrostimulation treatments. **g**, Measurements of root growth curvatures of single and double mutants of *arh1-1*, *fei1*, and *fei2* after hydrostimulation treatments for 24 hours. Each circle represents the data from an individual root. Boxplots span the first to the third quartiles of the data, and whiskers indicate the minimum and maximum values. The line in the box represents the mean. Scale bars represent 5 mm. “n” represents the number of roots analyzed in the experiment. Three biological replicates were carried out. One-way ANOVA with Tukey’s multiple comparison test was used for statistical analyses with *P* < 0.01.


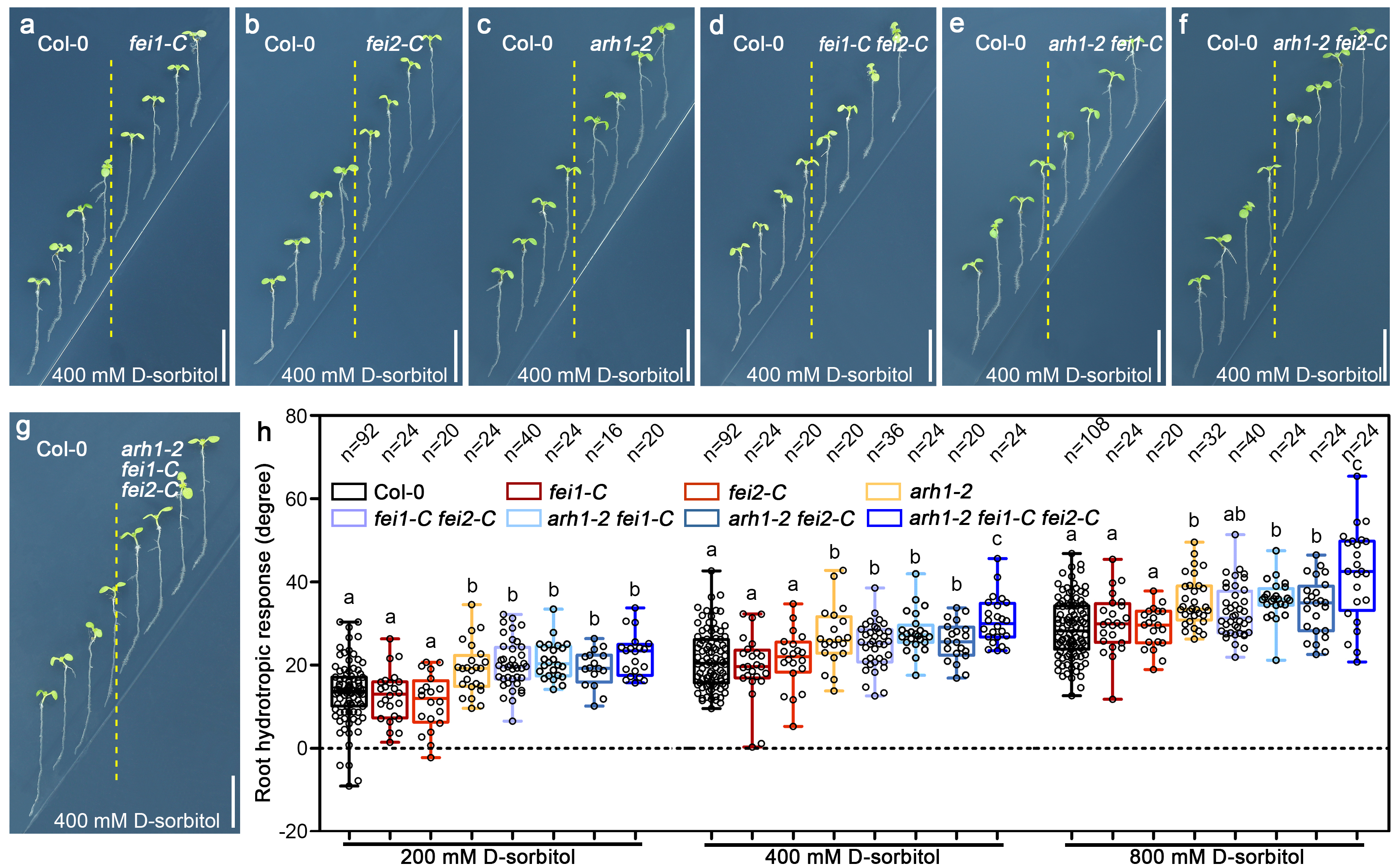


**Supplementary Figure 5 | Various single, double, and triple mutants of *ARH1*, *FEI1*, and *FEI2*, which were generated by gene editing, show enhanced response to hydrostimulation treatments.**

**a-g**, Hydrotropic response of representative wild-type (Col-0) and indicated mutants after hydrostimulation treatments. **h**, Measurements of root growth curvatures of single, double, and triple mutants of *arh1-2*, *fei1-C*, and *fei2-C* after hydrostimulation treatments for 24 hours. Boxplots span the first to the third quartiles of the data, and whiskers indicate the minimum and maximum values. The line in the box represents the mean. Each circle represents the data from an individual root. Scale bars represent 10 mm. “n” represents the number of roots analyzed in the experiment. Three biological replicates were carried out. One-way ANOVA with Tukey’s multiple comparison test was used for statistical analyses with *P* < 0.01.


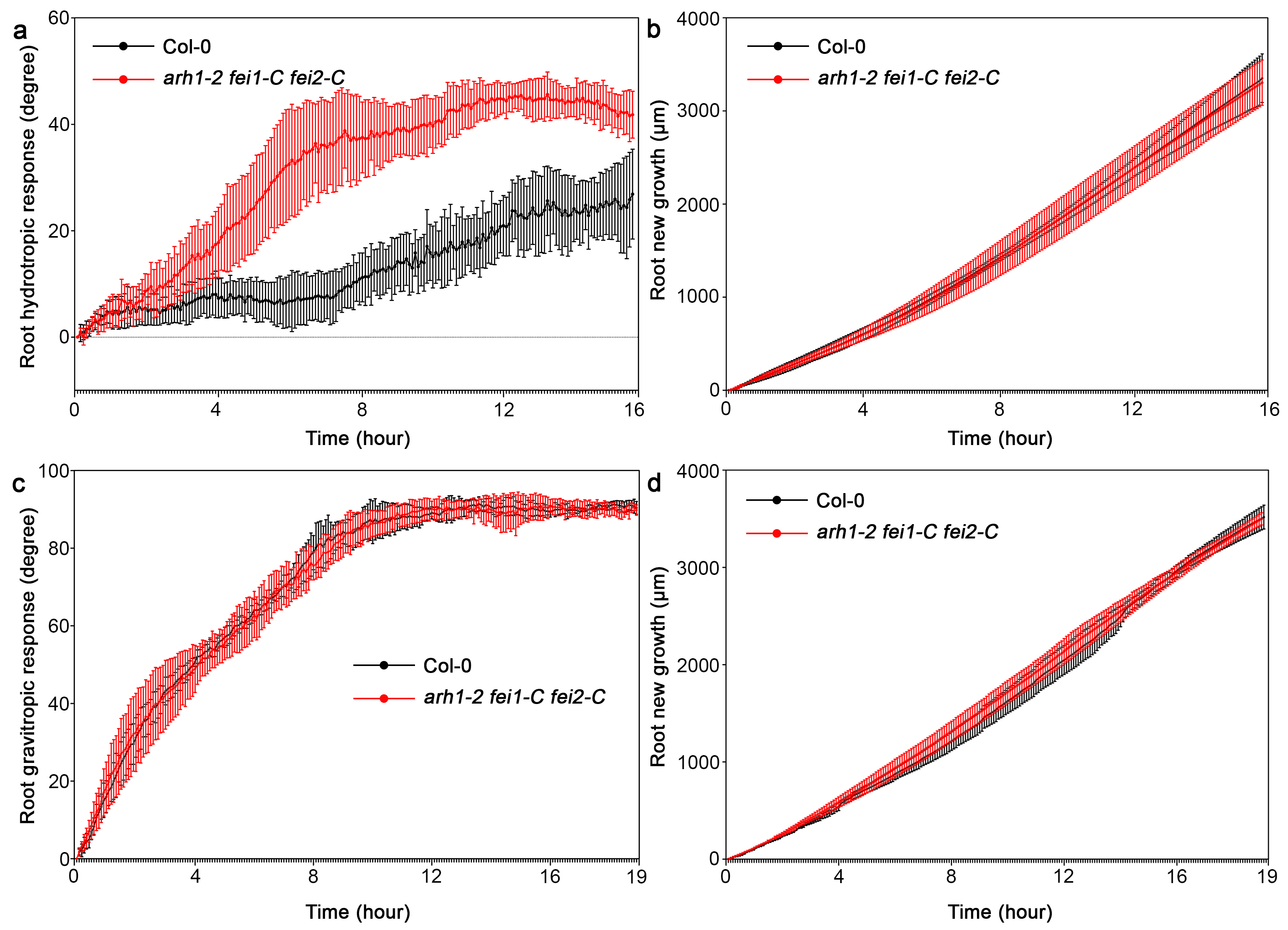


**Supplementary Figure 6 | The triple mutant roots exhibit an enhanced hydrotropic response while maintaining normal gravitropic response and growth rate compared with those of Col-0.**

**a-b**, Time course analyses of root hydrotropic responses and growth rates of Col-0 and the triple mutant. Four-day-old seedlings were transferred from 1/2 MS medium to hydrostimulating medium containing 400 mM D-sorbitol at the bottom right side of the plates. **c-d**, Time course analyses of gravitropic responses and growth rates of Col-0 and the triple mutant. Four-day-old seedlings were transferred from 1/2 MS medium to a new 1/2 MS medium and horizontally incubated. Four biological replicates were carried out.


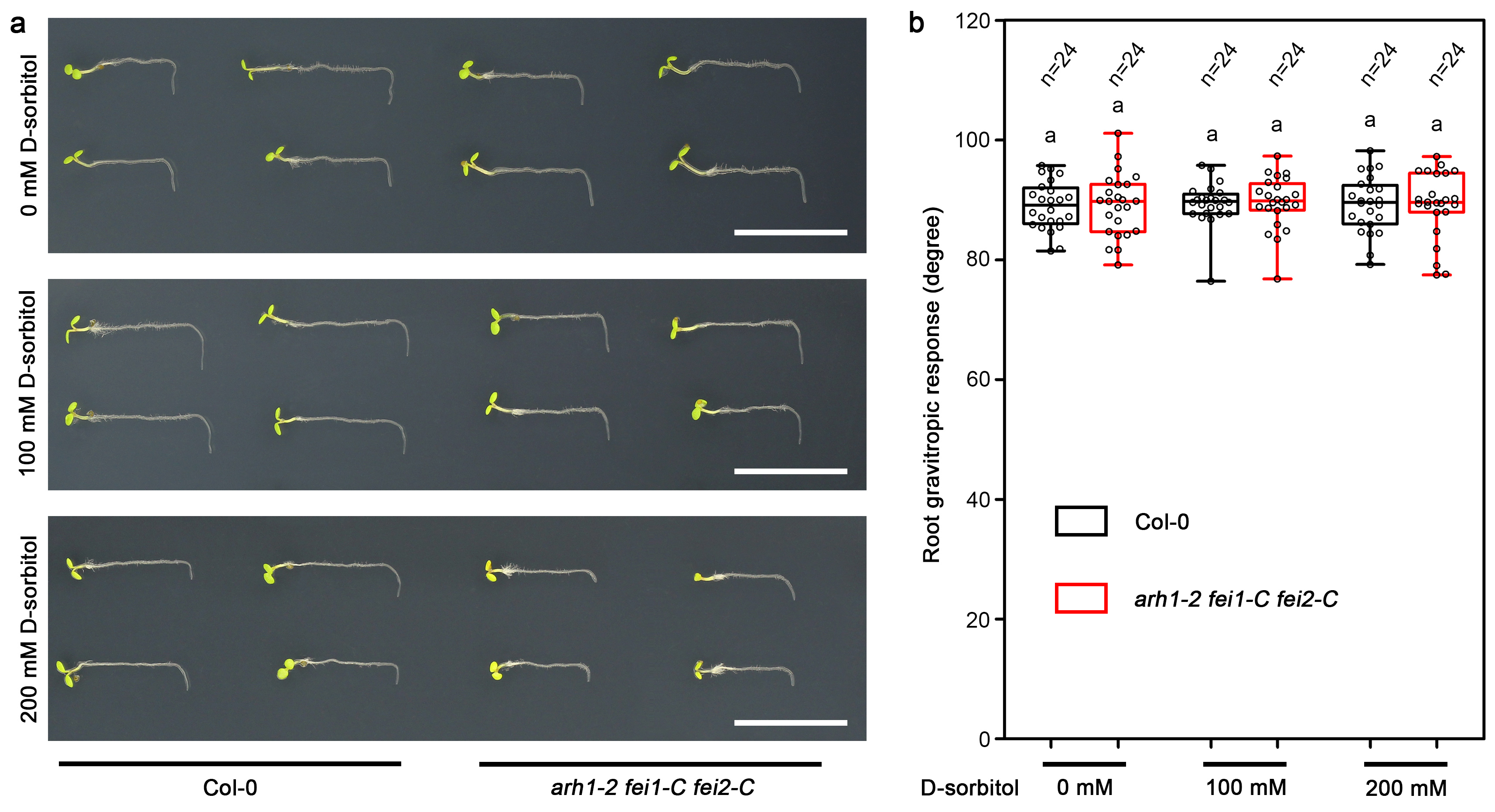


**Supplementary Figure 7 | Roots of the triple mutant *arh1-2 fei1-C fei2-C* showed a gravitropic response similar to those of Col-0 under various osmotic stresses.**

**a**, Gravitropic responses of Col-0 and the triple mutant. Four-day-old seedlings of Col-0 and the triple mutant were transferred from 1/2 MS medium to 1/2 MS medium supplemented with various concentrations of D-sorbitol and horizontally incubated for 24 hours. **b**, Measurements of root gravitropic curvatures after a 24-hour gravistimulation treatment under various osmotic stress conditions. Boxplots span the first to the third quartiles of the data, and whiskers indicate the minimum and maximum values. The line in the box represents the mean. Scale bars represent 10 mm. “n” represents the number of roots analyzed in the experiment. Three biological replicates were carried out. One-way ANOVA with Tukey’s multiple comparison test was used for statistical analyses with *P* < 0.01.

**
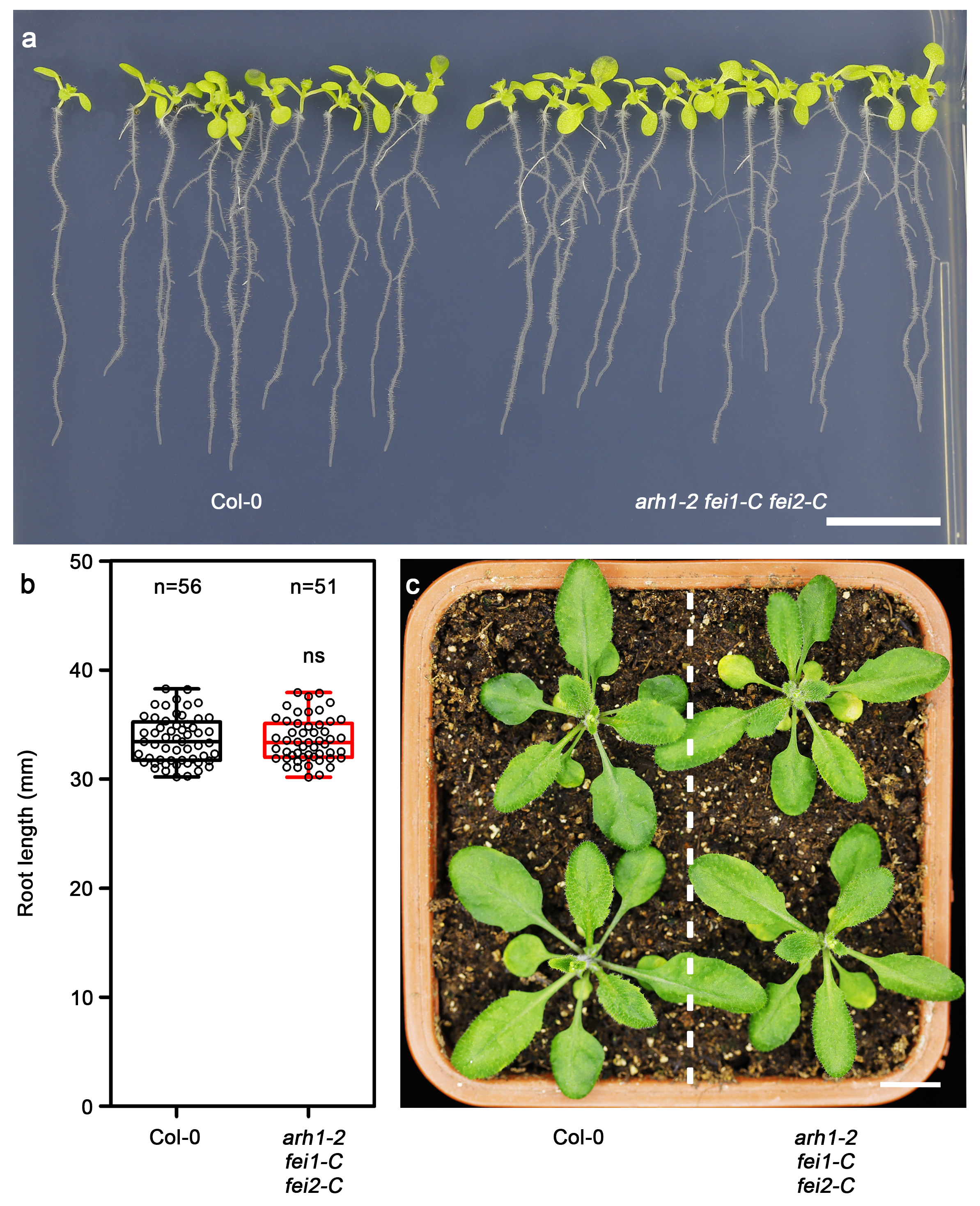
**

**Supplementary Figure 8 | The triple mutant shows no obvious growth defects compared with that of wild-type under normal growing conditions.**

**a**, Phenotypes of 10-day-old seedlings of Col-0 and *arh1-2 fei1-C fei2-C* grown on 1/2 MS medium. **b**, Root length of 10-day-old Col-0 and *arh1-2 fei1-C fei2-C* seedlings grown on 1/2 MS medium. **c**, Phenotypes of 30-day-old plants of Col-0 and *arh1-2 fei1-C fei2-C* grown in soil. Boxplots span the first to the third quartiles of the data, and whiskers indicate the minimum and maximum values. The line in the box represents the mean. Scale bars represent 10 mm. “n” represents the number of roots analyzed in the experiment. Three biological replicates were carried out. Statistical significance was determined by two-side and unpaired *t*-test, without making any adjustments for multiple comparisons (*P* < 0.01).


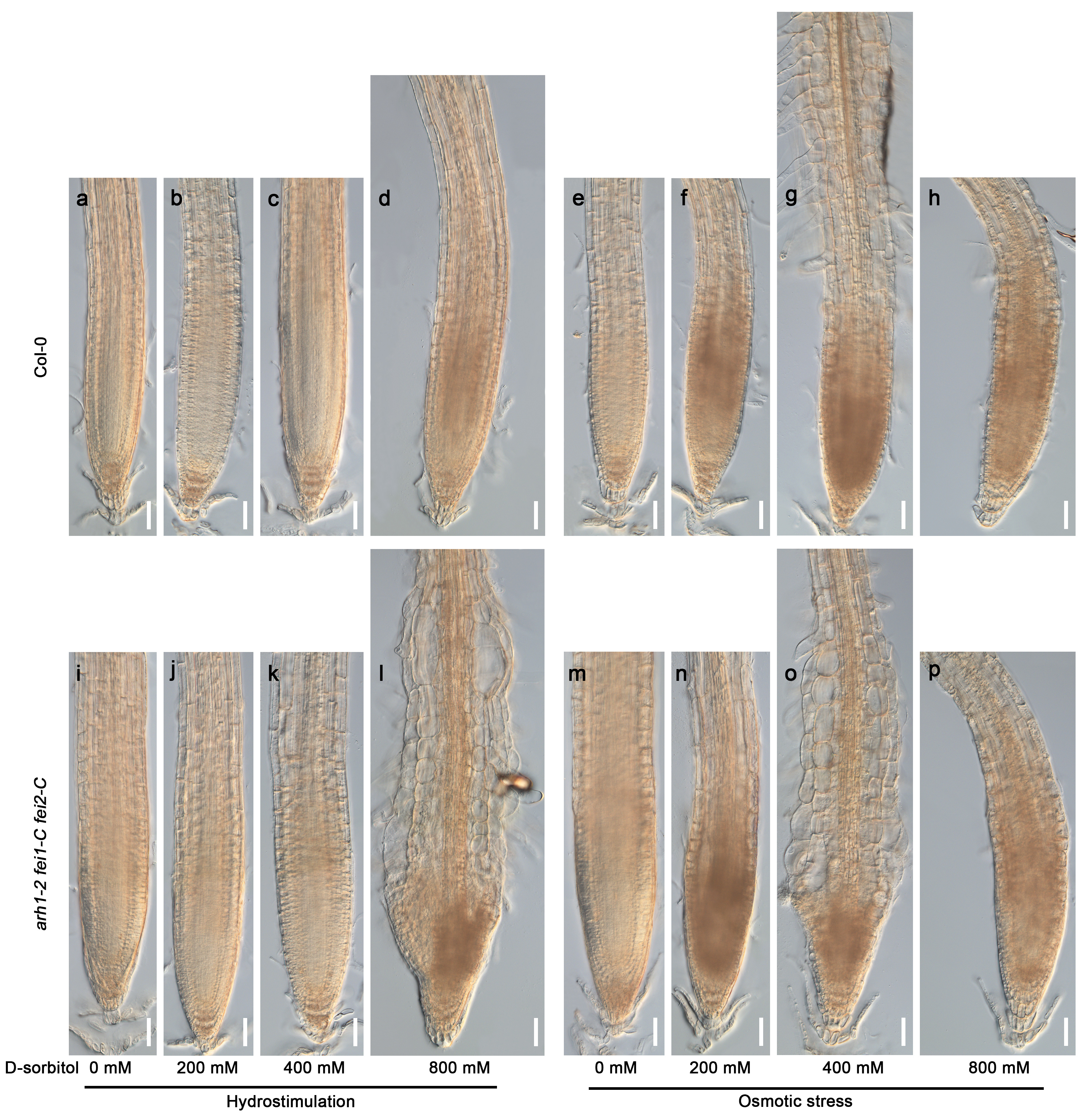


**Supplementary Figure 9 | Root tip cells of the triple mutants exhibited abnormal expansion upon hydrostimulation or osmotic stress treatment.**

**a**-**h**, Phenotypes in the root tips of Col-0 after treated with hydrostimulation or osmotic stress. Four-day-old Col-0 seedlings were transferred from 1/2 MS medium to split 1/2 MS media, supplemented with various concentrations of D-sorbitol at the bottom right side of the medium (a-d), or to osmotic stress media containing different concentrations of D-sorbitol (e-h) and incubated for 24 hours. **i-p**, Phenotypes in the root tips of the triple mutant after treated with hydrostimulation or osmotic stress. Four-day-old *arh1-2 fei1-C fei2-C* seedlings were transferred from 1/2 MS medium to split 1/2 MS media, supplemented with different concentrations of D-sorbitol at the bottom right side of the media (i-l) or to osmotic stress media containing different concentrations of D-sorbitol (m-p) and incubated for 24 hours. Three biological replicates were carried out. Scale bars represent 50 µm.


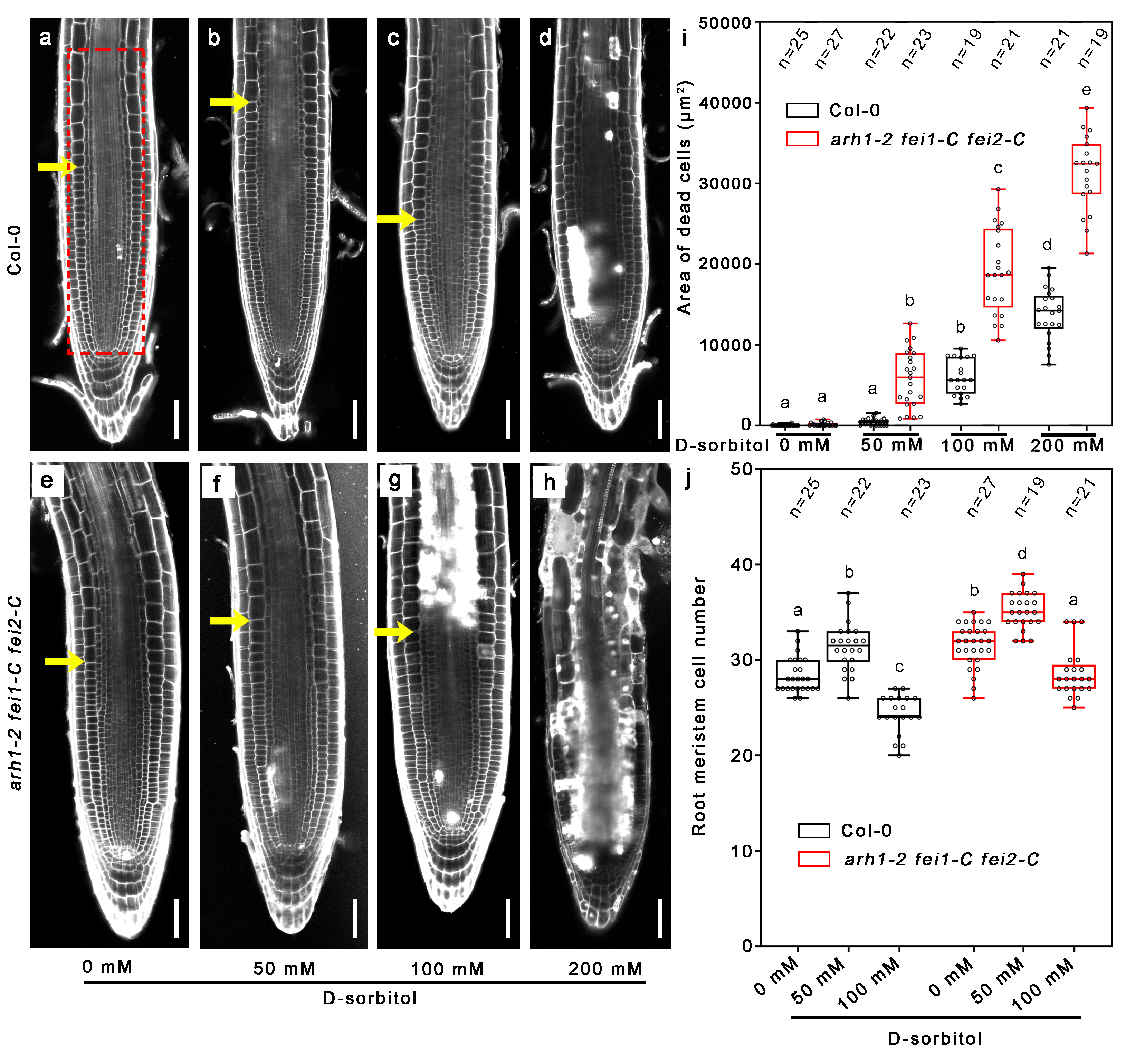


**Supplementary Figure 10 | Root tips of the triple mutant show reduced tolerance to** [**osmotic stress**](javascript:;)**.**

**a**-**h**, Representative propidium iodide-stained roots from four-day-old Col-0 and *arh1-2 fei1-C fei2-C* seedlings after transferred from 1/2 MS medium to 1/2 MS medium supplemented with 0 mM (a, e), 50 mM (b, f), 100 mM (c, g), or 200 mM (d, h) D-sorbitol, respectively, and incubated for 2 hours. **i**, Measurements of the dead cell areas in a 400 µm × 100 µm region (as shown in figure a) above the quiescent center. **j**, Cortex cell numbers in the meristematic zone of Col-0 and the triple mutant after osmotic stimulated for 2 hours. Yellow arrows shown in (a-c) and (e-g) mark the junction between meristematic and elongation zone. Scale bars represent 50 µm. Boxplots span the first to the third quartiles of the data, and whiskers indicate the minimum and maximum values. The line in the box represents the mean. Each circle represents the data from an individual root. “n” represents the number of roots analyzed in the experiment. Three biological replicates were carried out. One-way ANOVA with Tukey’s multiple comparison test was used for statistical analyses with *P* < 0.01.


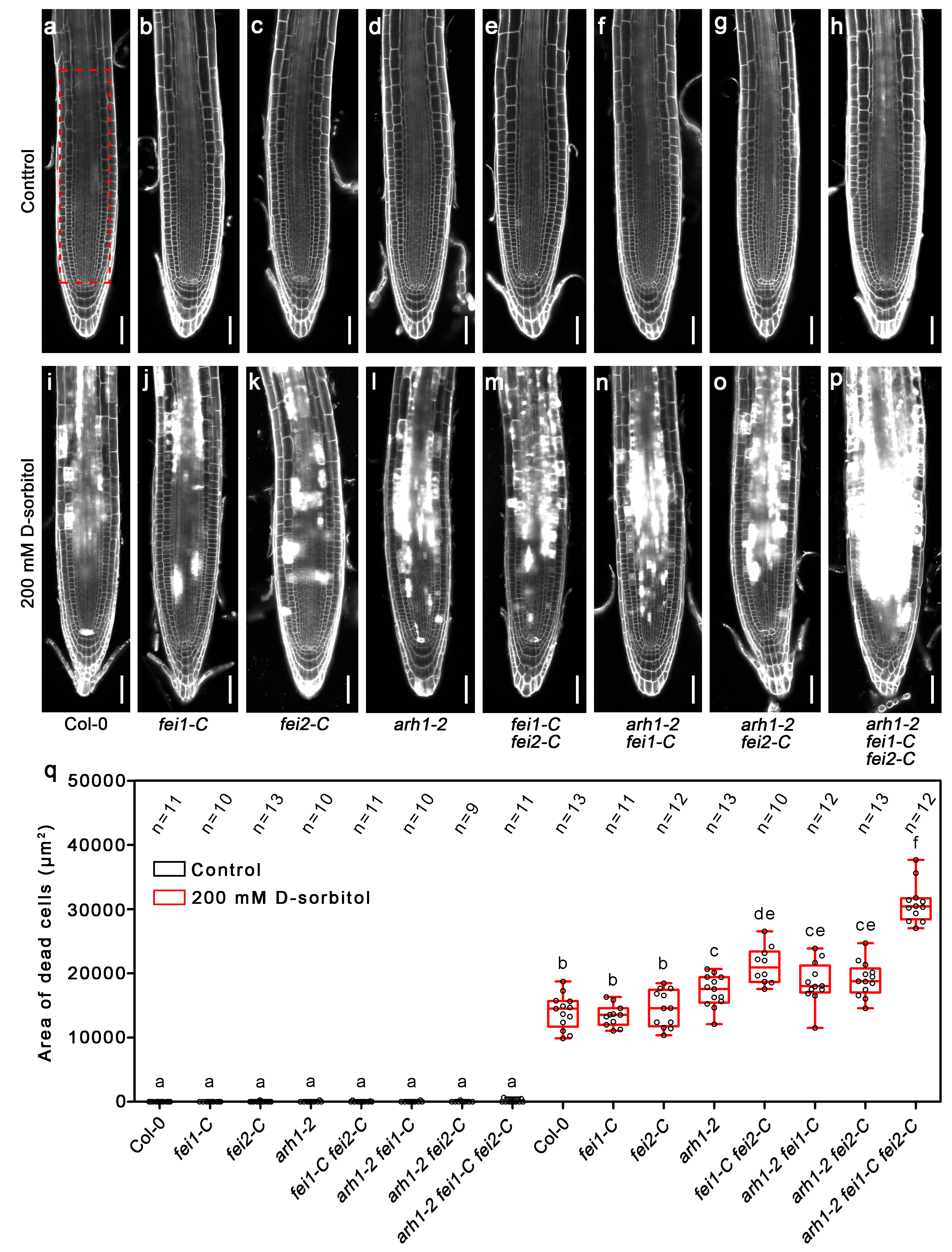


**Supplementary Figure 11 | Root tips of various single, double, and triple mutants of *ARH1*, *FEI1*, and *FEI2*, which were generated by gene editing, show reduced tolerance to osmotic stress.**

**a**-**p**, Representative propidium iodide-stained roots of four-day-old Col-0 and indicated mutants after transferred from 1/2 MS medium to 1/2 MS medium as controls (a-h), or to 1/2 MS medium supplemented with 200 mM D-sorbitol (i-p) and incubated for 2 hours. **q**, Measurements of the dead cell areas in a 400 µm × 100 µm region (as shown in figure a) above the quiescent center. Boxplots span the first to the third quartiles of the data, and whiskers indicate the minimum and maximum values. The line in the box represents the mean. Each circle represents the measurement of an individual root. Scale bars represent 50 µm. “n” represents the number of roots analyzed in the experiment. Three biological replicates were carried out. One-way ANOVA with Tukey’s multiple comparison test was used for statistical analyses with *P* < 0.01.


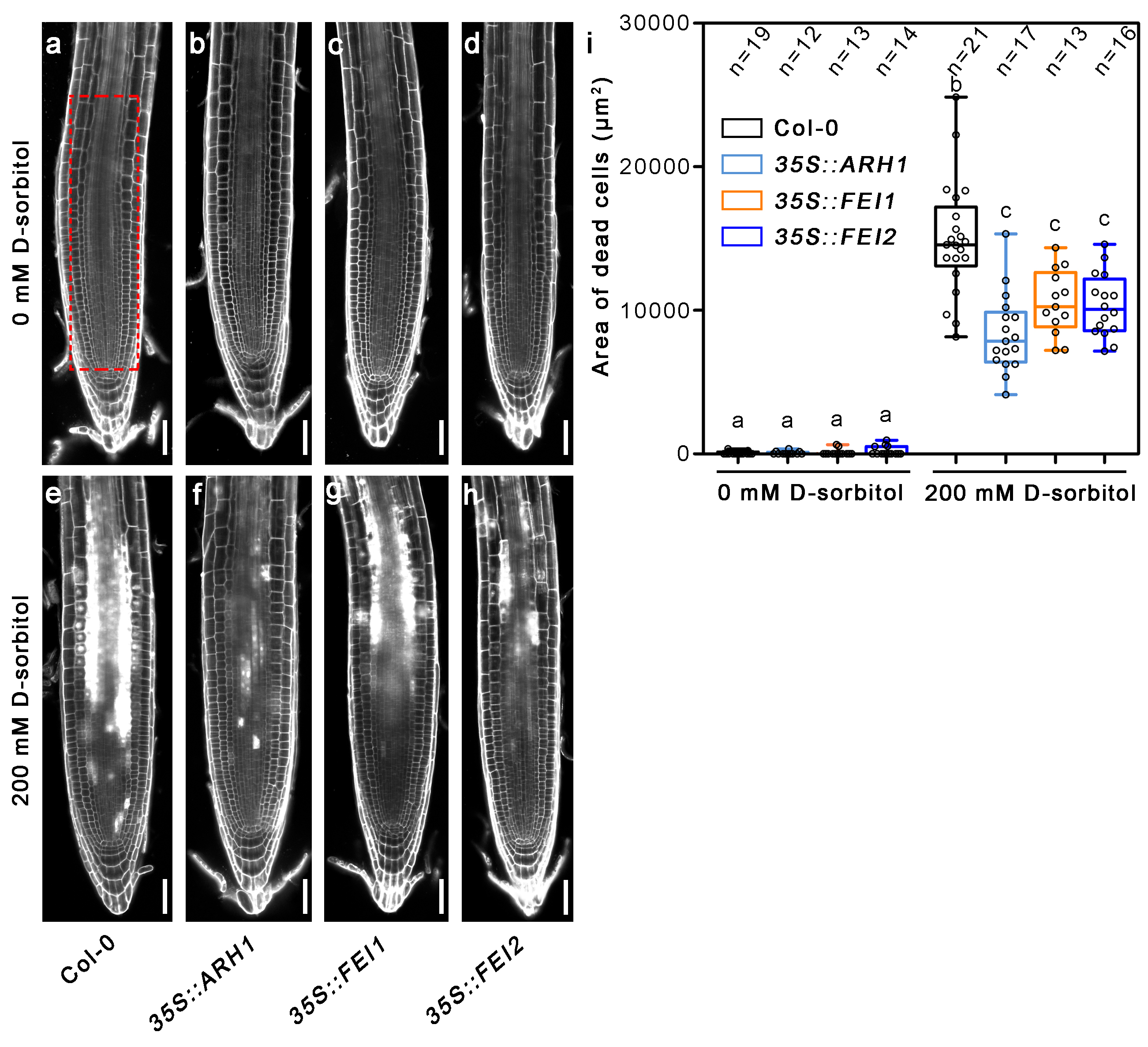


**Supplementary Figure 12 | Root tips of overexpression lines of *ARH1*, *FEI1*, and *FEI2* show enhanced tolerance to** [**osmotic stress**](javascript:;)**.**

**a**-**h**, Representative propidium iodide-stained roots from four-day-old Col-0, *35S::ARH1*, *35S::FEI1*, and *35S::FEI2* transgenic seedlings after transferred from 1/2 MS medium to 1/2 MS medium supplemented with 0 mM (a-d) or 200 mM (e-h) D-sorbitol, respectively, and incubated for 2 hours. **i**, Measurements of the dead cell areas in a 400 µm × 100 µm region (as shown in figure a) above the quiescent center. Scale bars represent 50 µm. Boxplots span the first to the third quartiles of the data, and whiskers indicate the minimum and maximum values. The line in the box represents the mean. Each circle represents the data from an individual root. “n” represents the number of roots analyzed in the experiment. Three biological replicates were carried out. One-way ANOVA with Tukey’s multiple comparison test was used for statistical analyses with *P* < 0.01.


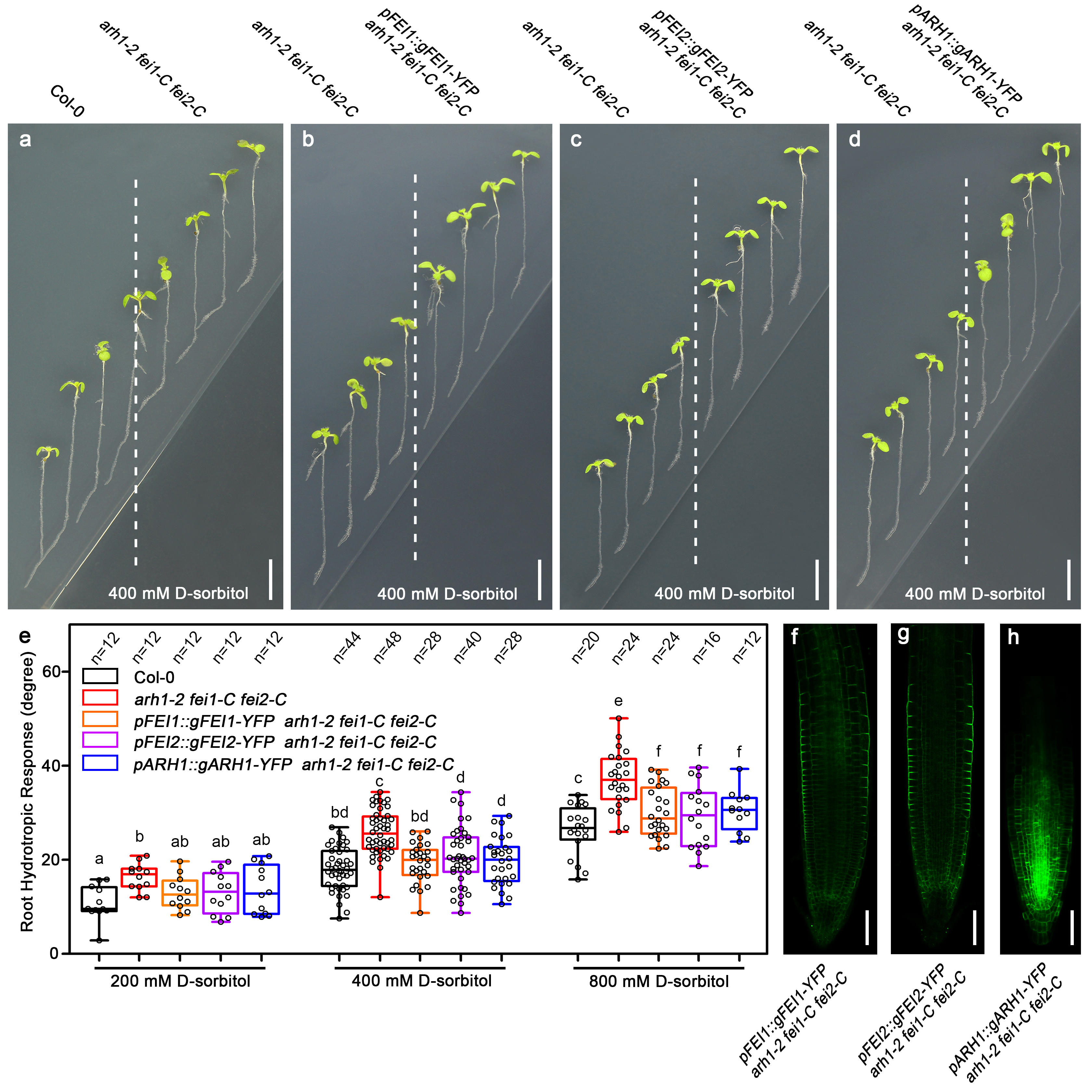


**Supplementary Figure 13 | Expression of *ARH1, FEI1,* or *FEI2* driven by their native promoters can rescue the enhanced root hydrotropic response of the triple mutant, *arh1-2 fei1-C fei2-C*.**

**a**-**d**, Hydrotropic response of representative wild-type (Col-0), *arh1-2 fei1-C fei2-C*, and the transgenic seedlings harboring *pARH1::gARH1-YFP*, *pFEI1::gFEI1-YFP*, or *pFEI2::gFEI2-YFP* in the *arh1-2 fei1-C fei2-C* background. **e**, Measurements of root hydrotropic curvatures of indicated seedlings after hydrostimulation treatments. **f**-**h**, Polar localizations of ARH1-YFP, FEI1-YFP, and FEI2-YFP in *arh1-2 fei1-C fei2-C* background were visualized in the primary root tips under a confocal microscope. Each circle represents the data from an individual root. Boxplots span the first to the third quartiles of the data, and whiskers indicate the minimum and maximum values. The line in the box represents the mean. Scale bars represent 10 mm in (a-d) and 50 µm in (f-h). “n” represents the number of roots analyzed in the experiment. All the experiments were performed for three biologically independent replications. One-way ANOVA with Tukey’s multiple comparison test was used for statistical analyses with *P* < 0.01.

**
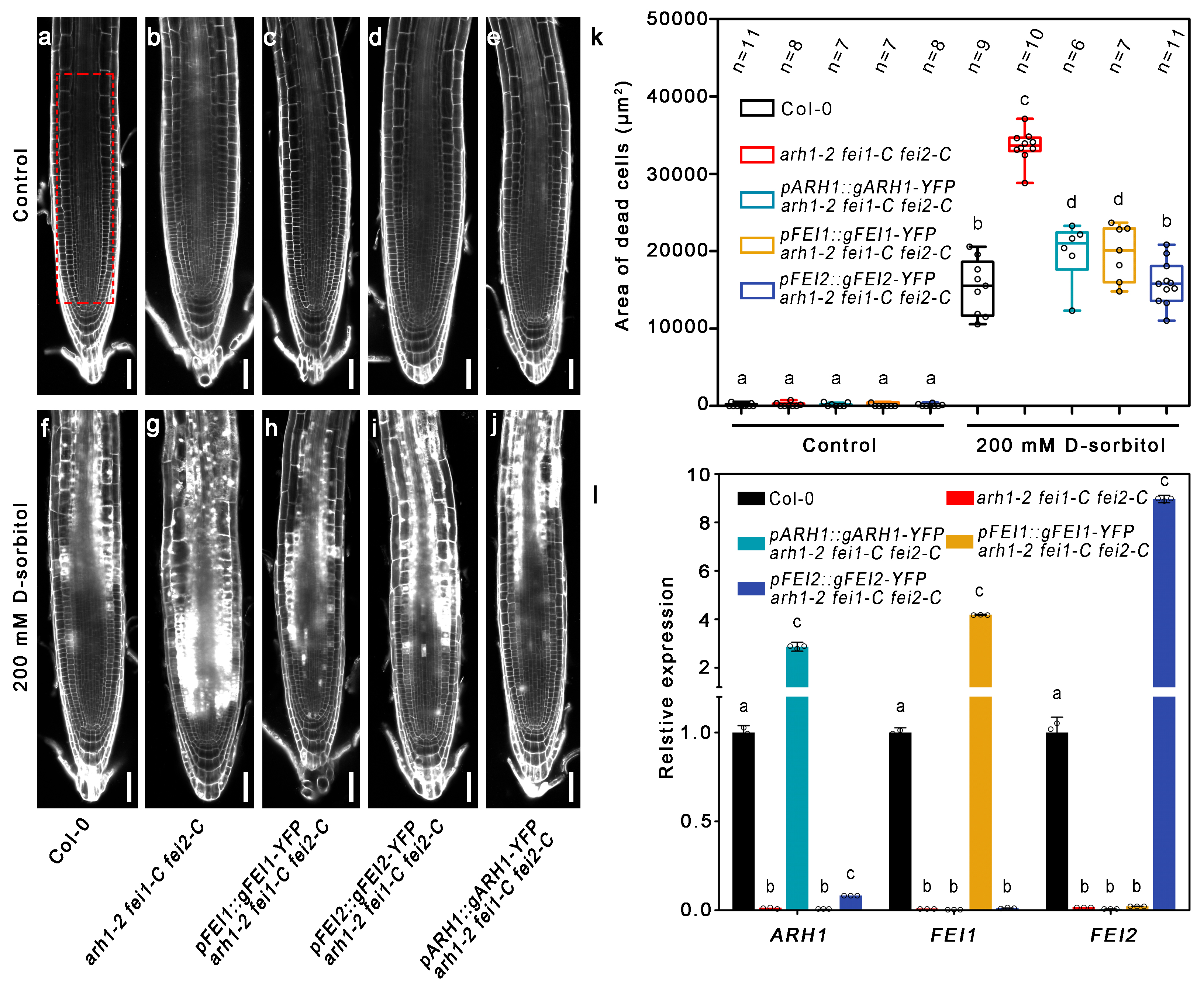
**

**Supplementary Figure 14 | Expression of *ARH1, FEI1,* or *FEI2* driven by their native promoters can rescue the reduced osmotic tolerance of the triple mutant, *arh1-2 fei1-C fei2-C*.**

**a**-**j**, Representative propidium iodide-stained roots from four-day-old Col-0, *arh1-2 fei1-C fei2-C*, and the transgenic seedlings harboring *pARH1::gARH1-YFP*, *pFEI1::gFEI1-YFP*, or *pFEI2::gFEI2-YFP* in *arh1-2 fei1-C fei2-C* background. Seedlings were transferred from 1/2 MS medium to 1/2 MS medium supplemented with 0 mM (a-e) or 200 mM (f-j) D-sorbitol, respectively, and incubated for 2 hours. **k**, Measurements of the dead cell areas in a 400 µm × 100 µm region (as shown in figure a) above the quiescent center. Boxplots span the first to the third quartiles of the data, and whiskers indicate the minimum and maximum values. The line in the box represents the mean. **l**, Relative expression levels of *ARH1*, *FEI1*, and *FEI2*, in the triple mutant and complemented transgenic plants. Data are the means ± SD of three biological replicates. Scale bars represent 50 µm. Each circle represents the data from an individual root (k) or sample (l). “n” represents the number of roots analyzed in the experiment. One-way ANOVA with Tukey’s multiple comparison test was used for statistical analyses with *P* < 0.01.


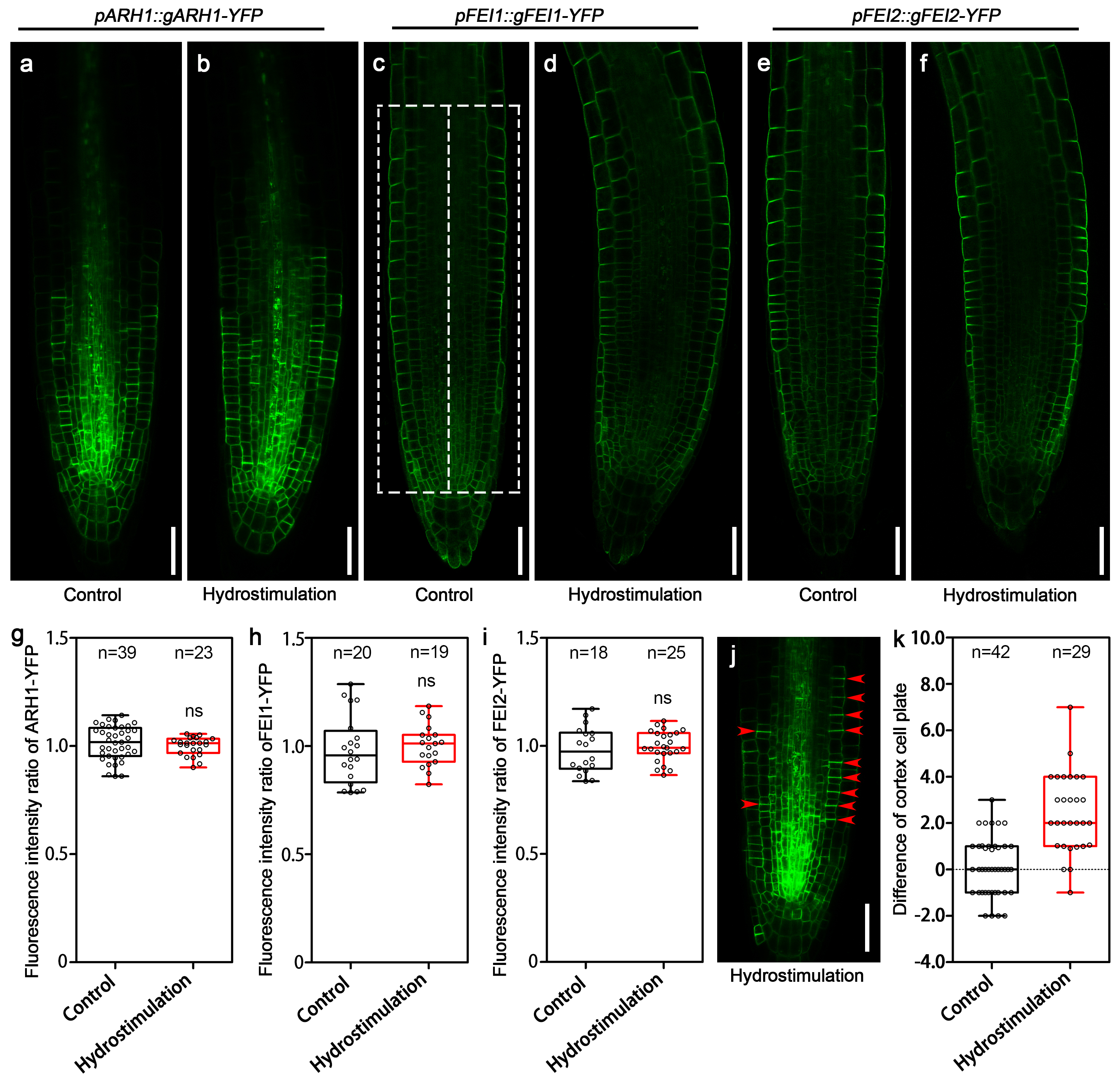


**Supplementary Figure 15 | The distributions and polar localizations of ARH1-YFP, FEI1-YFP, and FEI2-YFP were not altered after hydrostimulation treatment.**

**a**-**f**, The distributions and polar localizations of three LRR-RLKs before and after hydrostimulation treatment. Four-day-old transgenic seedlings harboring *pARH1::gARH1-YFP*, *pFEI1::gFEI1-YFP*, or *pFEI2::gFEI2-YFP* were treated with control (a, c, e) or hydrostimulation with 200 mM D-sorbitol at the bottom right side of the medium (b, d, f). **g**-**i**, YFP fluorescence ratio between the right and left sides (controls), or between convex and concave sides within a 400-μm root tip starting from the quiescent center (hydrostimulated seedlings) was analyzed (as depicted in figure c). **j**, A representative root tip of *pARH1::gARH1-YFP* after 2 hour hydrostimulation treatment, red arrows indicate the polar localization of *ARH1-YFP* facing to the newly formed cell plate. **k**, Differences of the number of newly formed cortical cell plates of right side versus left side (controls), or between convex and concave sides in the meristematic zone (hydrostimulated seedlings). Boxplots span the first to the third quartiles of the data, and whiskers indicate the minimum and maximum values. The line in the box represents the mean. Scale bars represent 50 µm. “n” represents the number of roots analyzed in the experiment. Three biological replicates were carried out. Statistical significance was determined by two-side and unpaired *t*-test, without making any adjustments for multiple comparisons (*P* < 0.01).

**
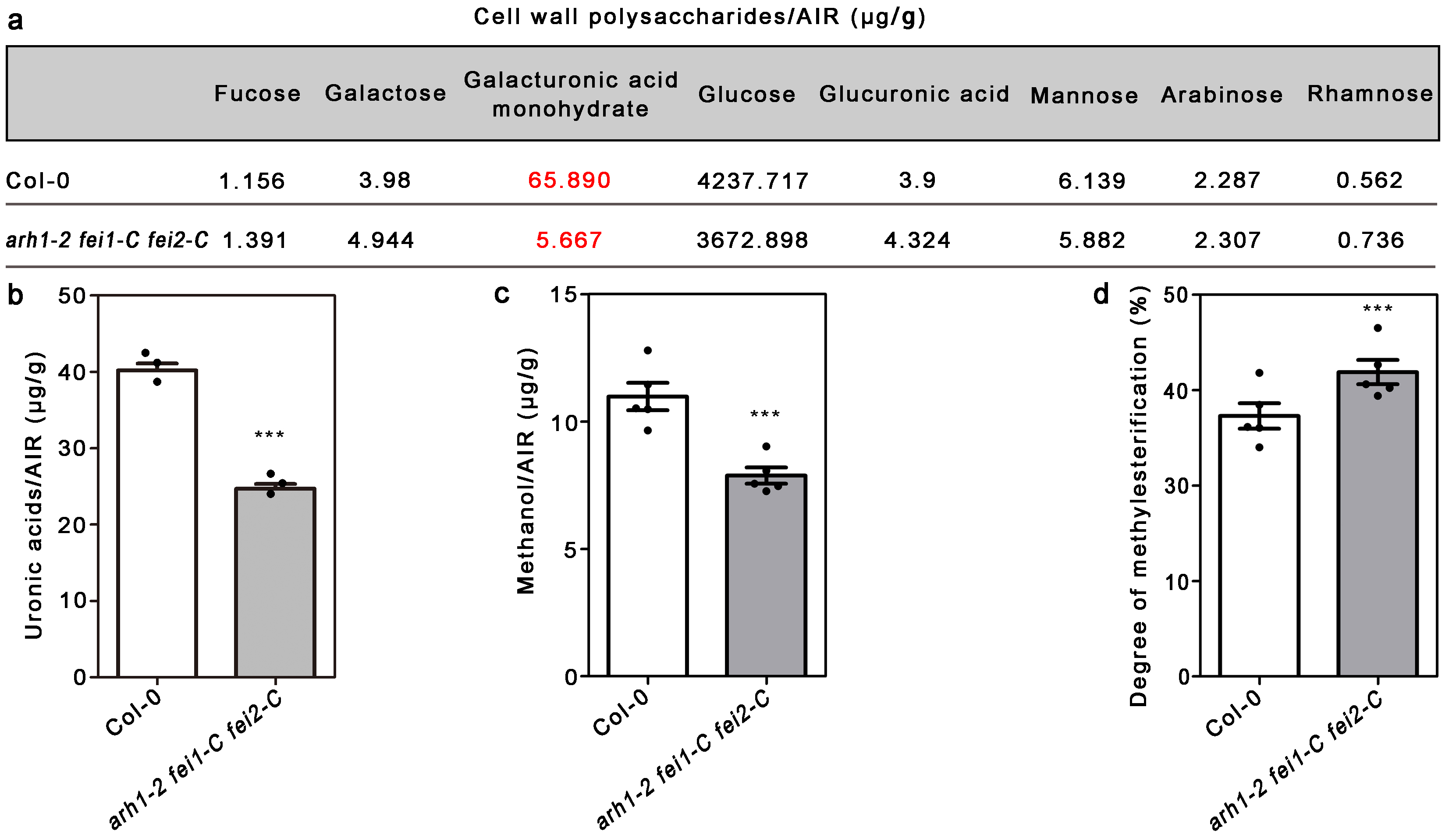
Supplementary Figure 16 | The amount of galacturonic acid monohydrate, one of the main pectin compositions, was significantly decreased in the root tips of the triple mutant.**

**a**, Levels of cell wall polysaccharides which were determined by GC-MS after the cell wall was isolated from the root tips of Col-0 and the triple mutant (polysaccharides/AIR, µg/g). Three biological replicates were carried out. **b**, Levels of uronic acids from the root tips of Col-0 and the triple mutant, which were tested by colorimetry and galacturonic acid used as reference. Data are the means ± SD of three biological replicates. **c**, Methanol released from the cell wall of the root tips of Col-0 and the triple mutant. Five biological replicates were carried out. **d**, Percentage of pectin methylesterification from the cell wall of the root tips of Col-0 and the triple mutant. Data are the means ± SD of five biological replicates. Statistical significance was determined by two-side and unpaired *t*-test, without making any adjustments for multiple comparisons (*P* < 0.01).


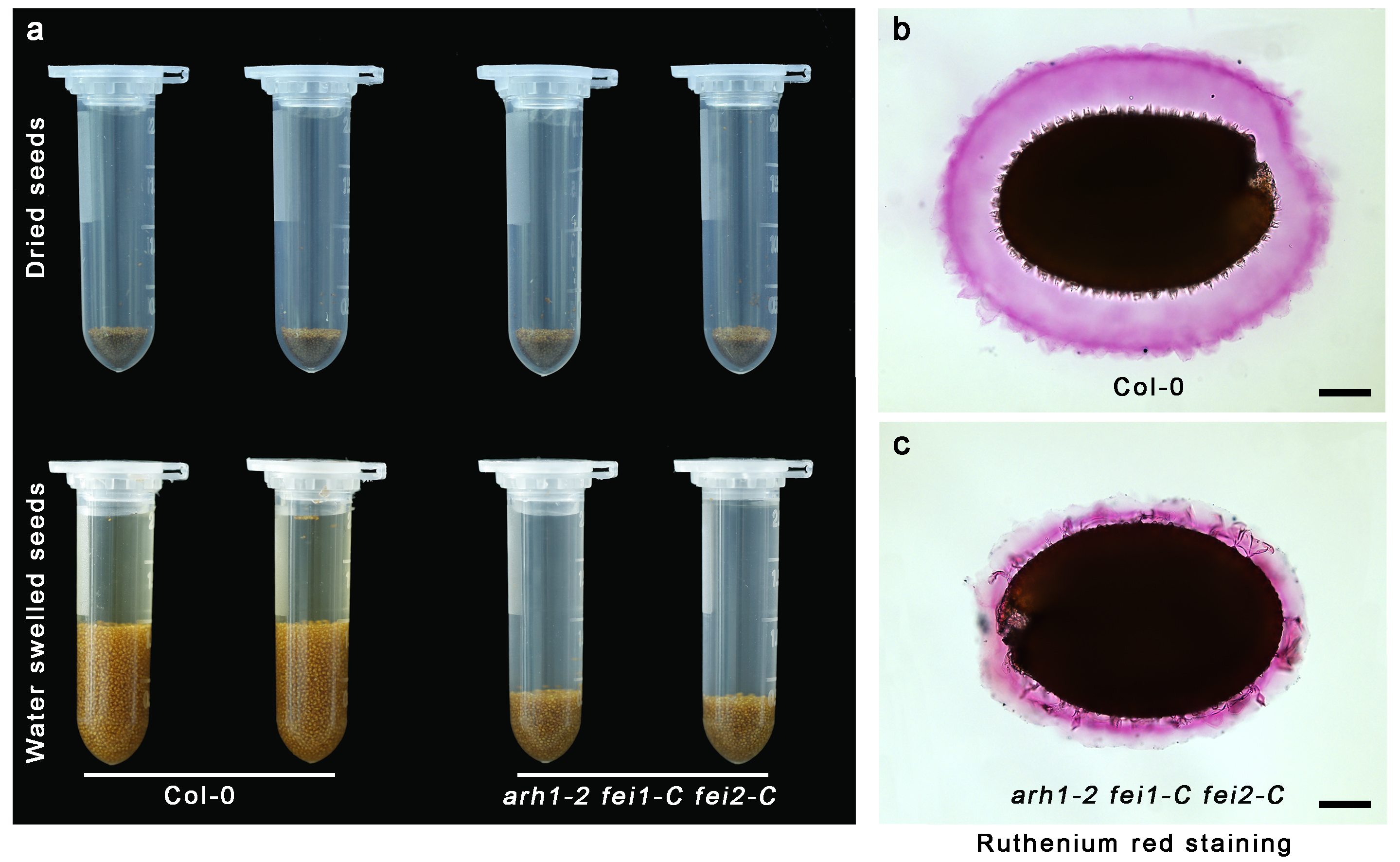


**Supplementary Figure 17 | The triple mutant displays defects in the seed coat mucilage.**

**a**, Volumes of 100 mg dried and 24-hour water-soaked seeds of wild type (Col-0) and *arh1-2 fei1-C fei2-C*. **b**, **c**, Seeds of Col-0 and *arh1-2 fei1-C fei2-C* were stained with 0.01% Ruthenium red for 10 min to show their seed coat mucilage. Three biological replicates were carried out. Scale bars represent 50 µm in (b) and (c).


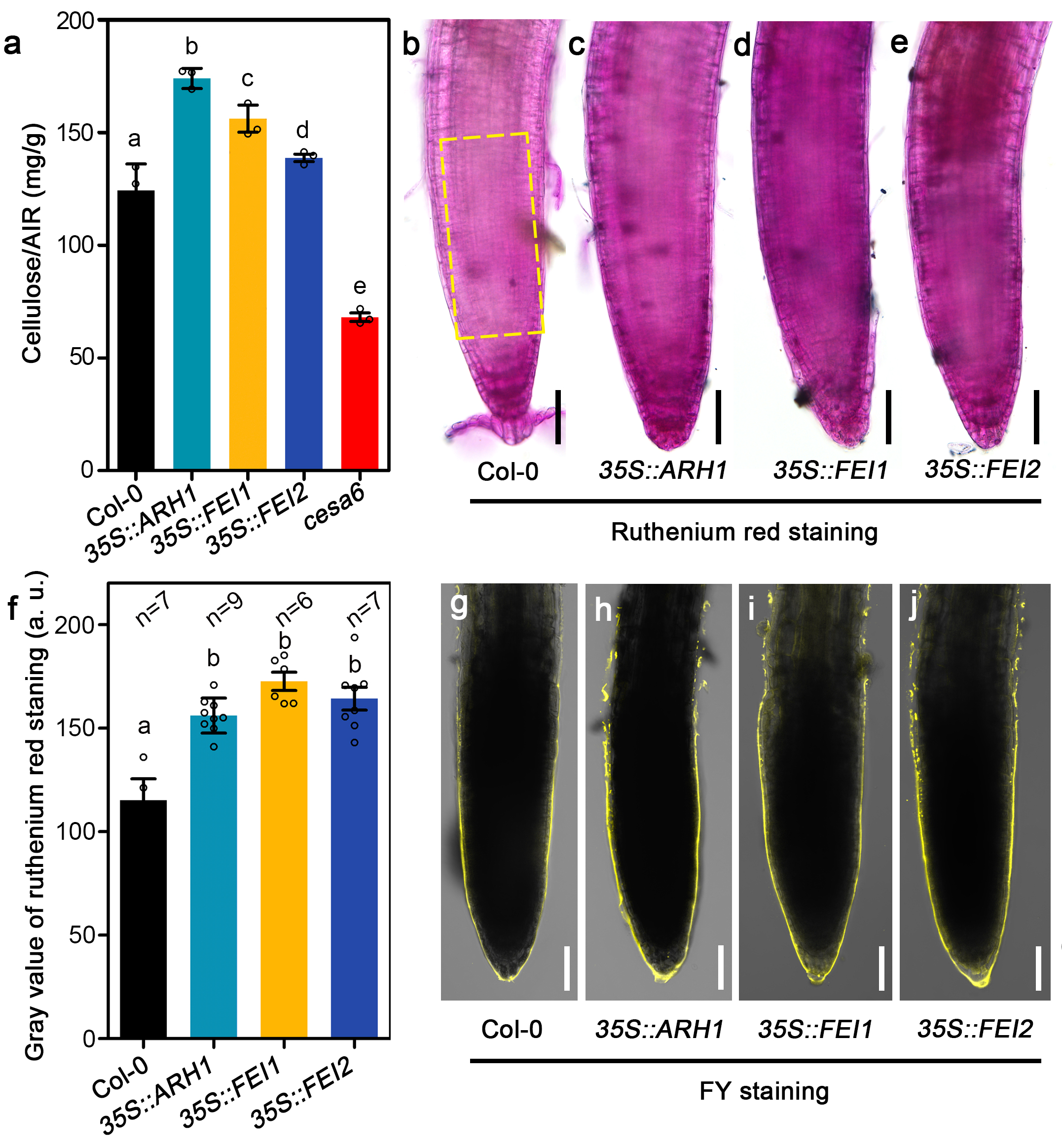


**Supplementary Figure 18 | Cell wall and its depositions were thickened in the overexpression lines of *ARH1*, *FEI1*, and *FEI2*.**

**a**, The amount of cellulose was measured from cell wall, defined as alcohol-insoluble residues (AIR), in the root tips of Col-0, *35S::ARH1*, *35S::FEI1*and *35S::FEI2* transgenic plants*,* as well as *cesa6* mutant. Three biological replicates were carried out. **b**-**e**, Ruthenium red stained root tips from four-day-old Col-0 (b), and transgenic lines of *35S::ARH1* (c), *35S::FEI1* (d), and *35S::FEI2* (e) seedlings. **f**, Relative staining intensity for the root tips as represented in (b-e) in a 200 µm × 100 µm area (as shown in b). **g**-**h**, Fluorol yellow (FY) stained root tips from seedlings of two-day-old Col-0, and transgenic lines of *35S::ARH1* (h), *35S::FEI1* (i), and *35S::FEI2* (j) seedlings. Three biological replicates were carried out. Scale bars represent 50 µm in (b-e and g-h). Each circle represents the measurement of an individual sample. “n” represents the number of roots analyzed in the experiment. Data are the means ± SD of three biological replicates. One-way ANOVA with Tukey’s multiple comparison test was used for statistical analyses with *P* < 0.01.


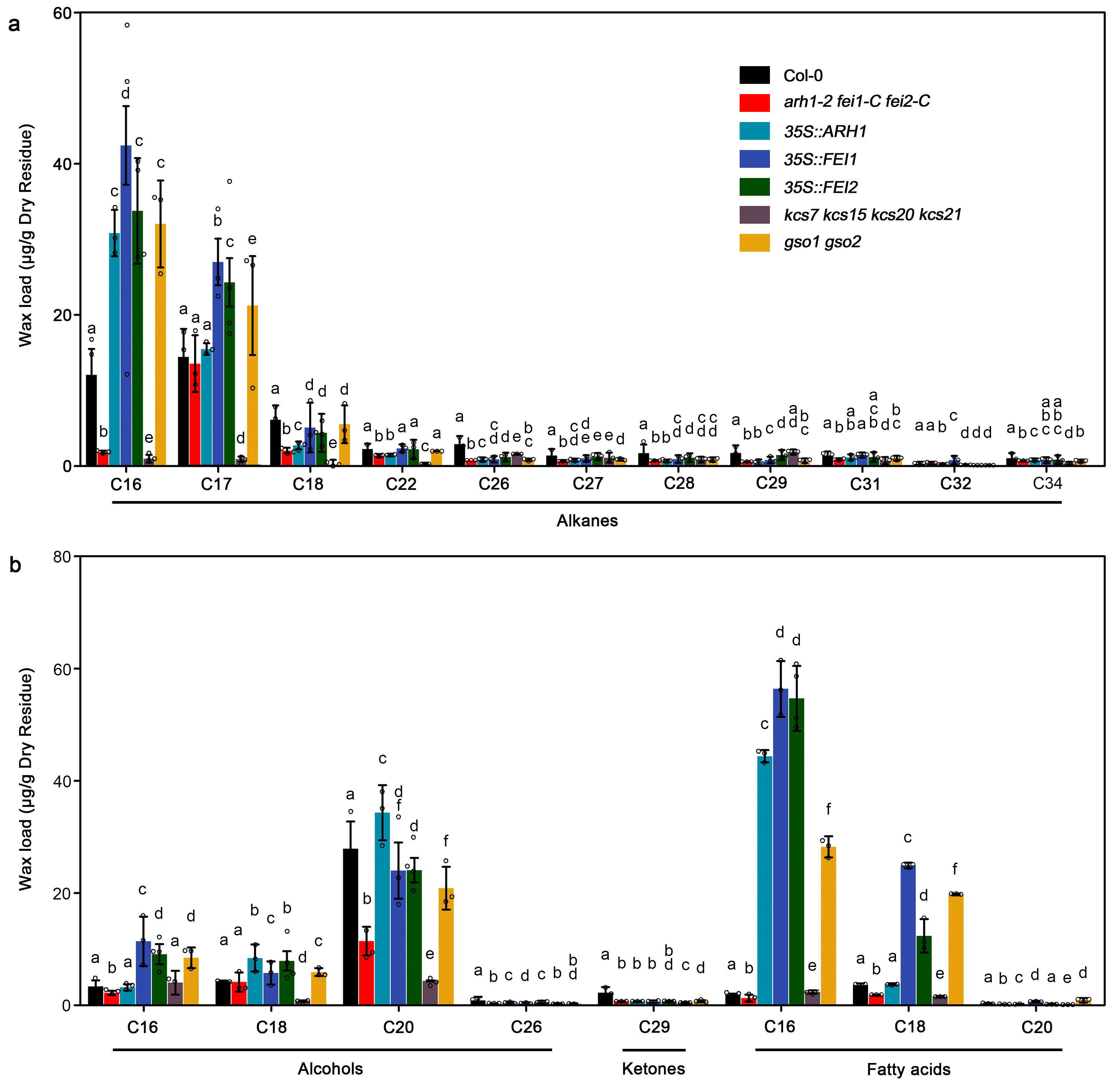


**Supplementary Figure 19 | The compositions and amounts of root cap wax.**

**a**, Carbon-chain-length distributions of alkanes in wax extracted from the 5-mm-long root tips of Col-0 and other genotypes. **b**, Carbon-chain-length distributions of alcohols, ketones and fatty acids in wax extracted from the 5-mm-long root tips of Col-0 and other genotypes. Data are the means ± SD of three biological replicates. Each circle represents the measurement of an individual sample. One-way ANOVA with Tukey’s multiple comparison test was used for statistical analyses with *P* < 0.01.


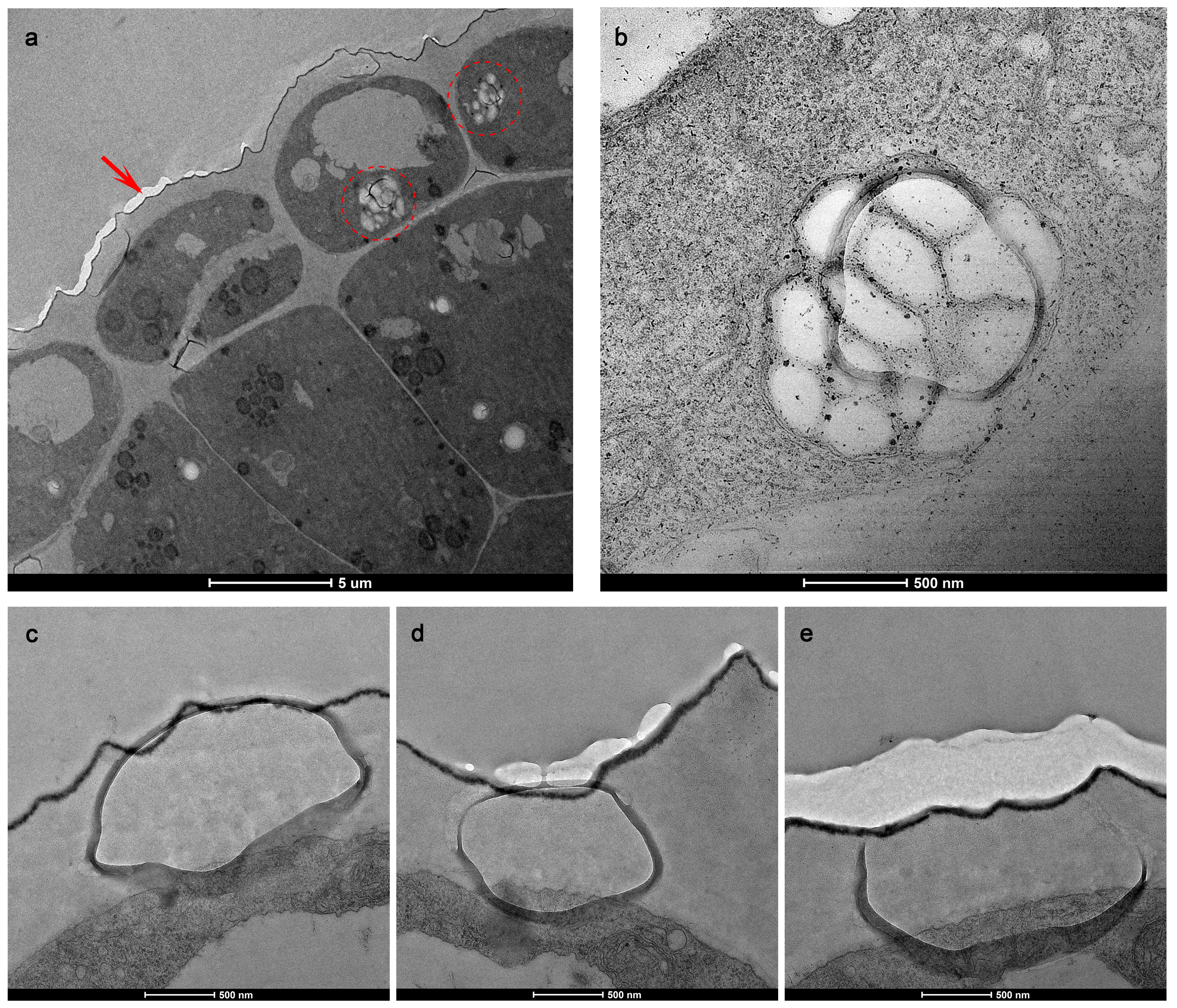


**Supplementary Figure 20 | Wax is first accumulated in the cytoplasm of the outmost cells of the root cap and then transported to the out layer of cutin.**

**a**, The root cap wax is apparently accumulated in the cytoplasm and later transported to the outermost cell layer of the root caps, as revealed by transmission electron microscopy (TEM) analysis. The red arrow indicates the depositions of wax on the outer surface of the cutin. Dot cycles indicate the wax drop in the cytoplasm of lateral root cap cells. **b**, The drop of wax was observed in the cytoplasm of the lateral root cap cells. **c-e**, The drop of wax is transported from the lateral root cap cells to the outside layer of cutin. Three biological replicates were carried out. Scale bars represent 5 µm in (a) and 500 nm in (b-e).

**
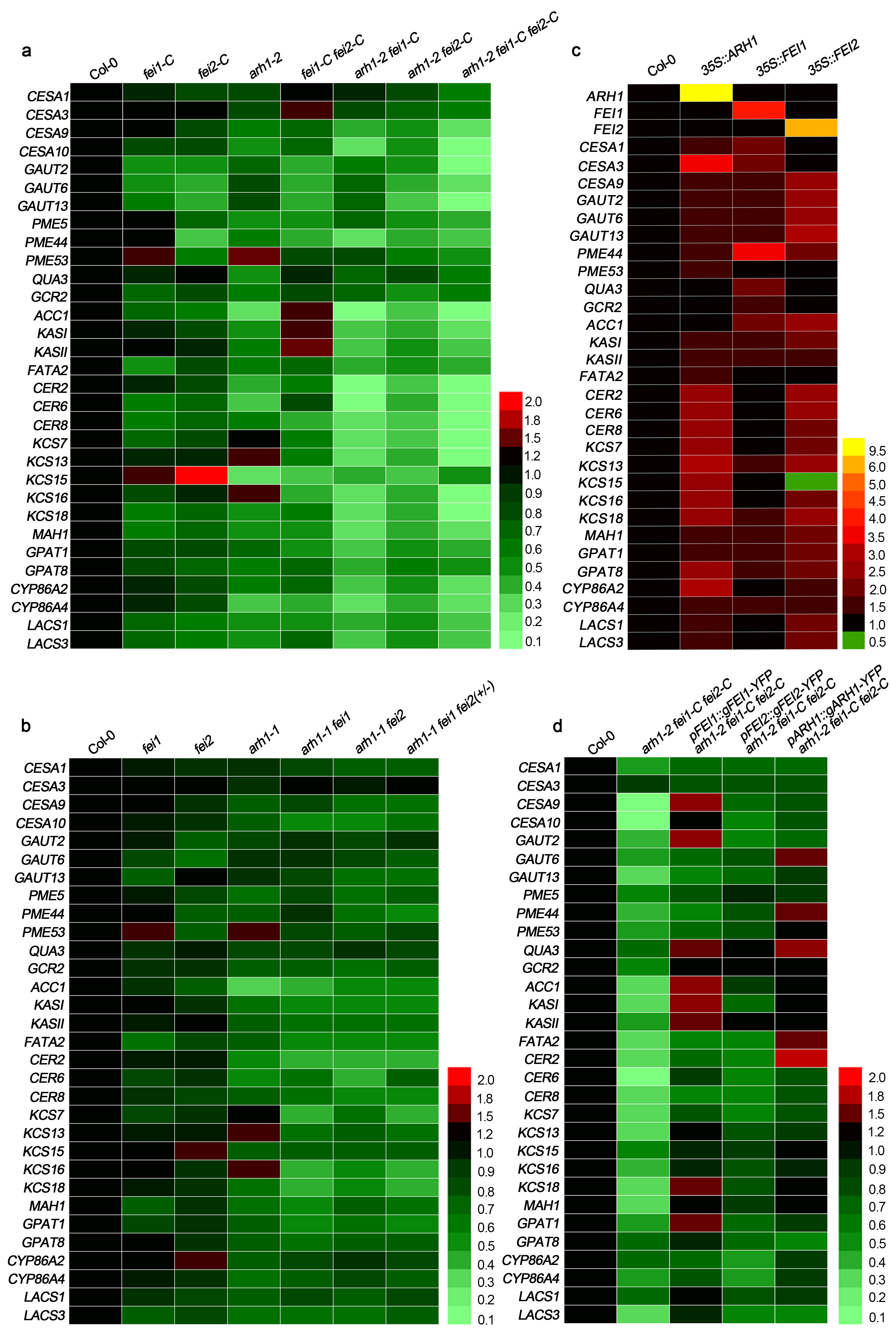
**

**Supplementary Figure 21 | Transcriptional levels of genes encoding for enzymes catalyzing the biosynthesis of CCW in various mutants, and overexpression lines of *ARH1*, *FEI1*, and *FEI2*.**

**a, b**, RT-qPCR analyses show the expression levels of genes in root tips of single, double, and triple mutants of *ARH1*, *FEI1*, and *FEI2,* generated by gene editing (a) or T -DNA insertions (b). **c**, Gene expression levels in the root tips of overexpression lines of *ARH1*, *FEI1*, and *FEI2*. **d**, Gene expression levels in the root tips of transgenic seedlings harboring *pARH1::gARH1-YFP*, *pFEI1::gFEI1-YFP*, or *pFEI2::gFEI2-YFP* in *arh1-2 fei1-C fei2-C* background. Data are the means of three biological replicates.

**
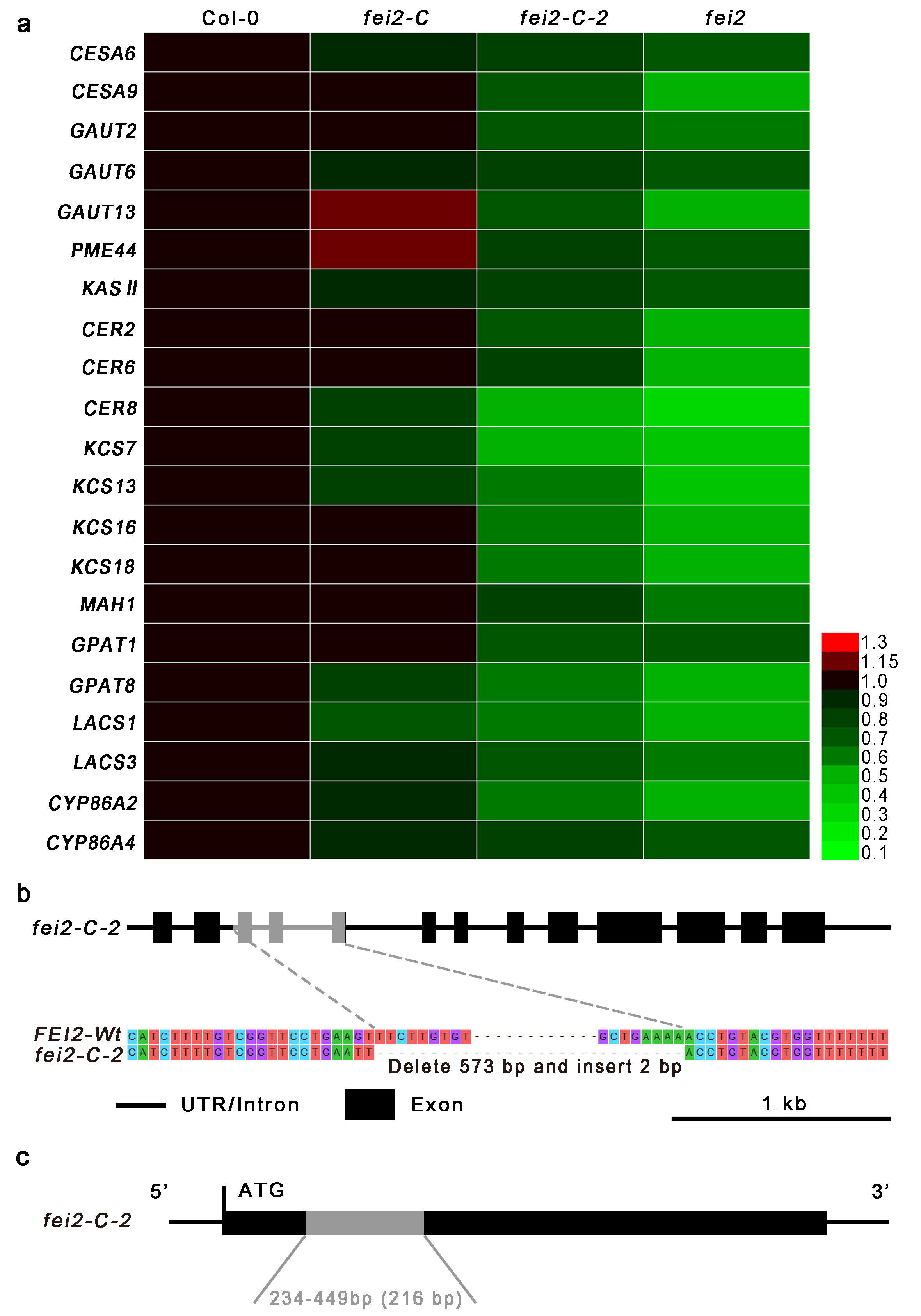
**

**Supplementary Figure 22 | Transcriptional levels of genes encoding for enzymes catalyzing the biosynthesis of CCW in various mutants of *FEI2*.**

**a**, RT-qPCR analyses show the expression levels of the genes in the root tips of *FEI2* mutants. *fei2-C* and *fei2-C-2* were generated through gene editing, and *fei2* is a T-DNA insertion line of *FEI2*. **b**, Gene editing details at the genomic sequence level of *fei2-C-2*. The deleted sequences were marked in gray. **c**, Gene editing details at cDNA level of *fei2-C-2*. Data are the means of three biological replicates.


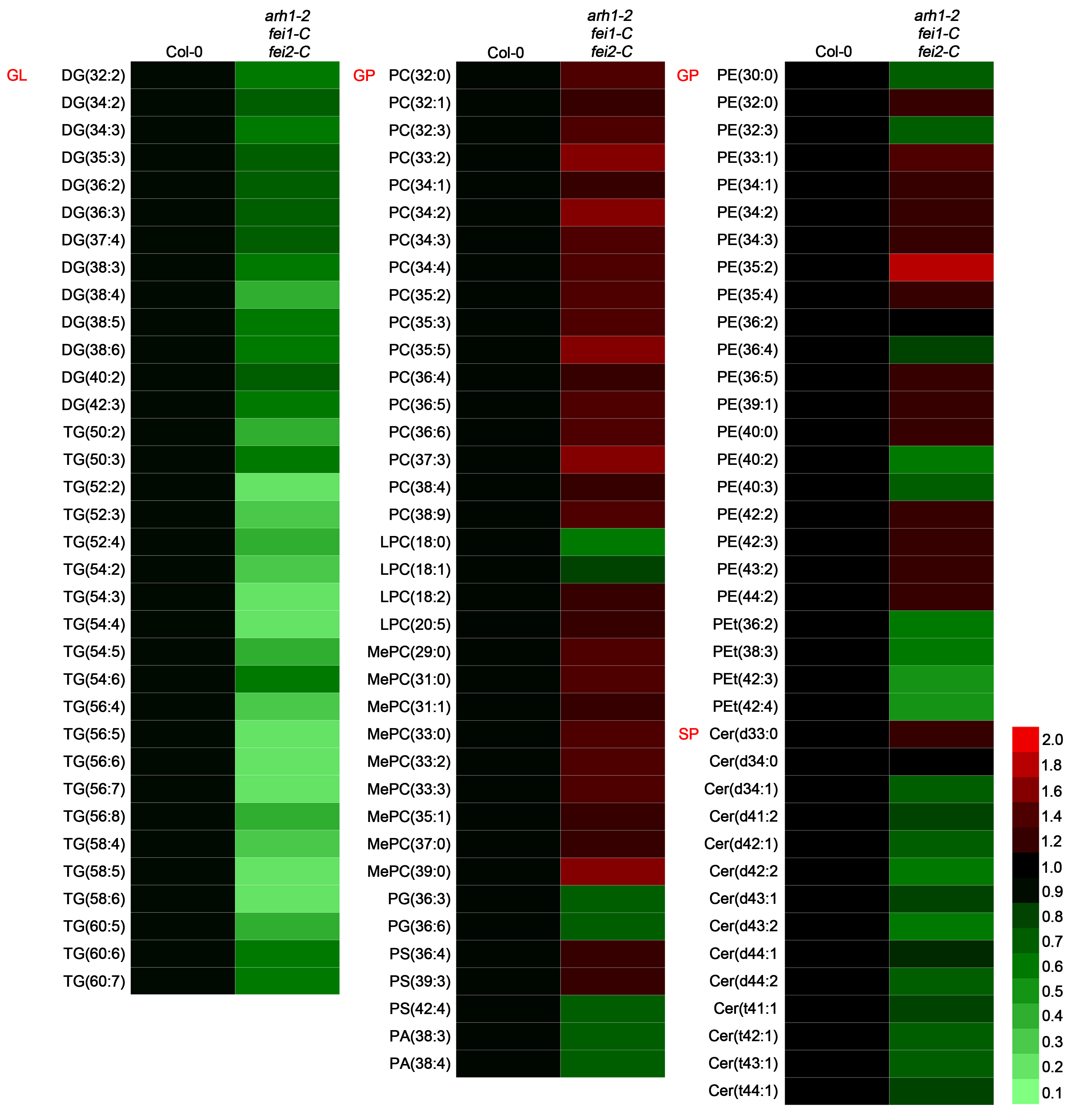


**Supplementary Figure 23 | ARH1, FEI1, and FEI2 modulate lipid metabolism in root tips.**

Relative amount of lipids in 5-mm root tips of wild-type (Col-0) and *arh1-2 fei1-C fei2-C*, which are presented by heatmaps according to the lipidomic analysis (n=6), *P*<0.05. GL, glycerolipids; DG, diglyceride; TG, triglyeride; SL, saccharolipids; GP, glycerophospholipids; PC, phosphatidylcholine; LPC, lyso-phosphatidylcholine; MePC, methylphosphatidylcholine; PG, phosphatidylglycerol; PS, phosphatidylserine; PA, phosphatidic acid; PE, phosphatidylethanolamine; PEt, phosphatidylethanol; SP, sphingolipids; Cer, ceramides. Data are the means of six biological replicates.


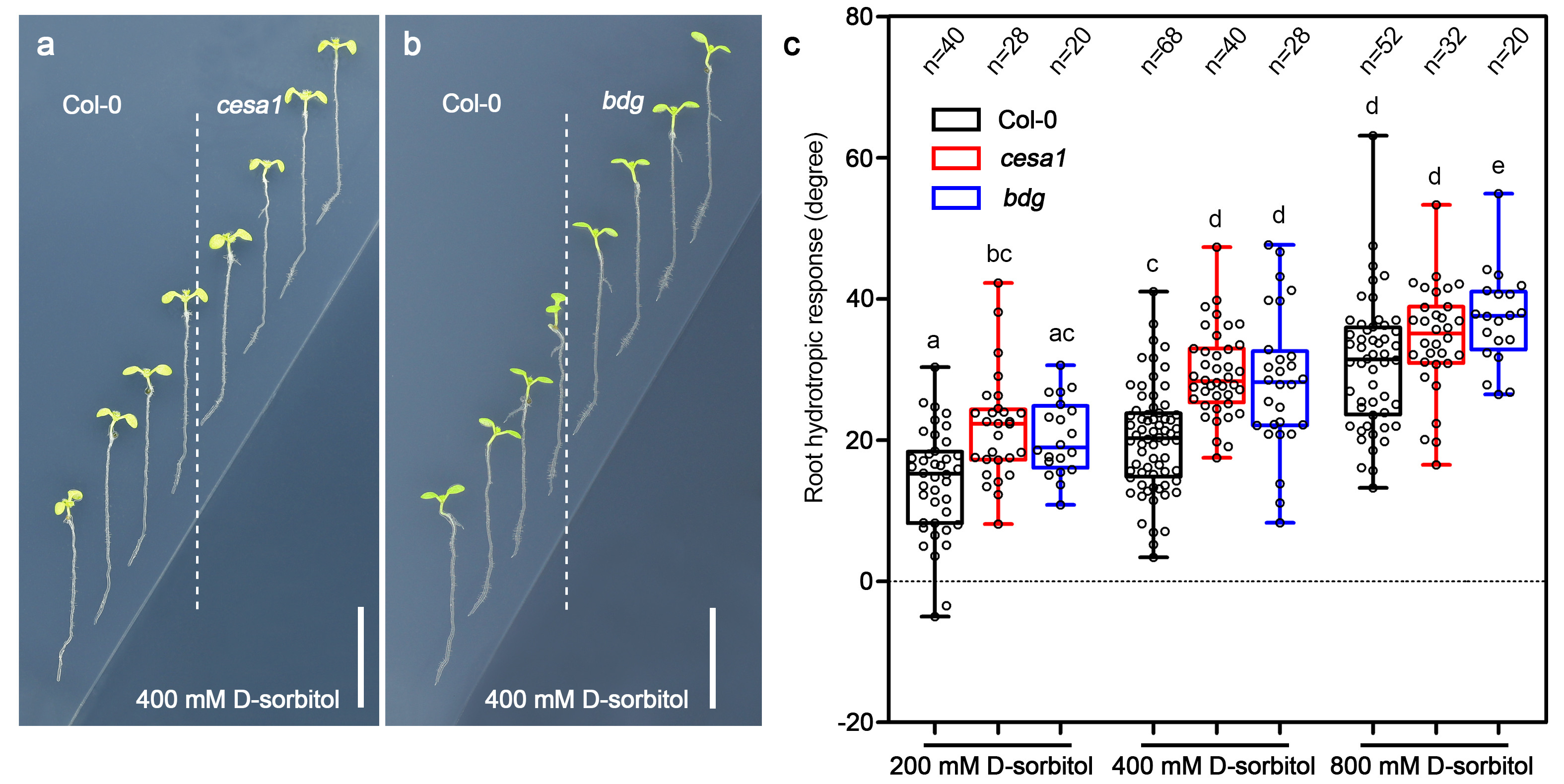


**Supplementary Figure 24 | Mutants with defects in cell wall or cutin show enhanced response to a moisture gradient.**

**a**, **b**, Hydrotropic responses of wild-type (Col-0) and mutants with defects in cell wall (cellulose) and cutin. **c**, Root growth curvatures of *cesa1* and *bdg* after 24-hour hydrostimulation treatments. Boxplots span the first to the third quartiles of the data, and whiskers indicate the minimum and maximum values. The line in the box represents the mean. Each circle represents the measurement of an individual root. “n” represents the number of roots analyzed in the experiment. Three biological replicates were carried out. One-way ANOVA with Tukey’s multiple comparison test was used for statistical analyses with *P* < 0.01.


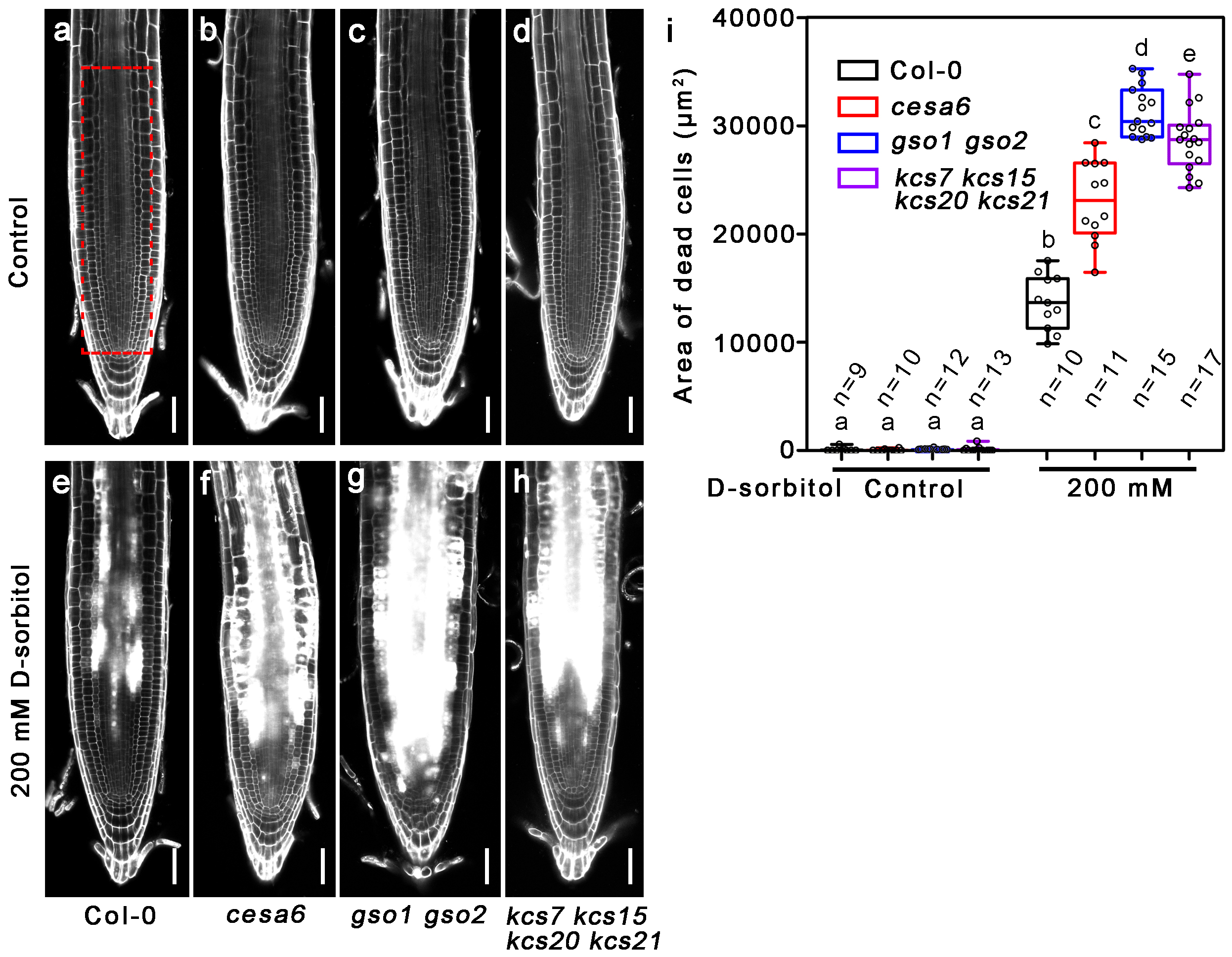


**Supplementary Figure 25 | Mutants with defects in CCW all showed reduced osmotic tolerance.**

**a**-**h**, Representative propidium iodide-stained root tips of Col-0 and various mutants after transferred from 1/2 MS medium to 1/2 MS medium as control (a-d) or to 1/2 MS medium supplemented with 200 mM D-sorbitol (e-h) and incubated for 2 hours. **i**, Measurements of the dead cell areas in a 400 µm × 100 µm region (as shown in figure a) above the quiescent center. Scale bars represent 50 µm. Boxplots span the first to the third quartiles of the data, and whiskers indicate the minimum and maximum values. The line in the box represents the mean. Each circle represents the measurement of an individual root. “n” represents the number of roots analyzed in the experiment. Three biological replicates were carried out. One-way ANOVA with Tukey’s multiple comparison test was used for statistical analyses with *P* < 0.01.


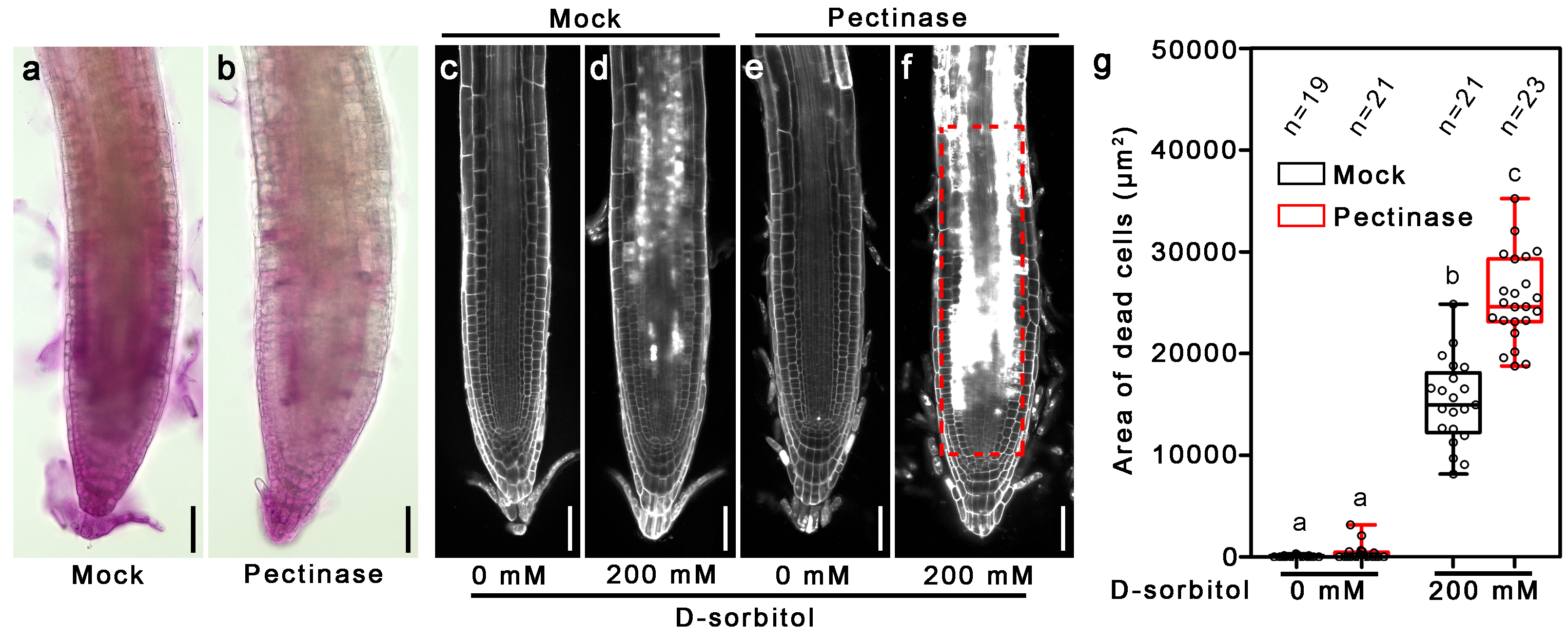


**Supplementary Figure 26 | Roots treated with pectinase show reduced tolerance to osmotic stress.**

**a**, **b**, *In vitro* pectinase treated Col-0 roots were stained with 0.05% Ruthenium red for 15 min and visualized under a microscope. **c**, **d**, Three-day-old Col-0 seedlings were transferred to 1/2 MS medium containing DMSO and incubated for 24 hours, then they were transferred to 1/2 MS medium containing 0 mM (c) or 200 mM (d) D-sorbitol and incubated for 2 hours. **e**, **f**, Three-day-old Col-0 seedlings were transferred to 1/2 MS medium containing 50 µM pectinase (dissolved in DMSO) and treated for 24 hours, then the seedlings were transferred to 1/2 MS medium with 0 mM (e) or 200 mM (f) D-sorbitol and incubated for 2 hours. **g**, Measurements of the dead cell areas in a 400 µm × 100 µm region (as shown in figure f) above the quiescent center. Scale bars represent 50 µm. Boxplots span the first to the third quartiles of the data, and whiskers indicate the minimum and maximum values. The line in the box represents the mean. Each circle represents the measurement of an individual root. “n” represents the number of roots analyzed in the experiment. Three biological replicates were carried out. One-way ANOVA with Tukey’s multiple comparison test was used for statistical analyses with *P* < 0.01.


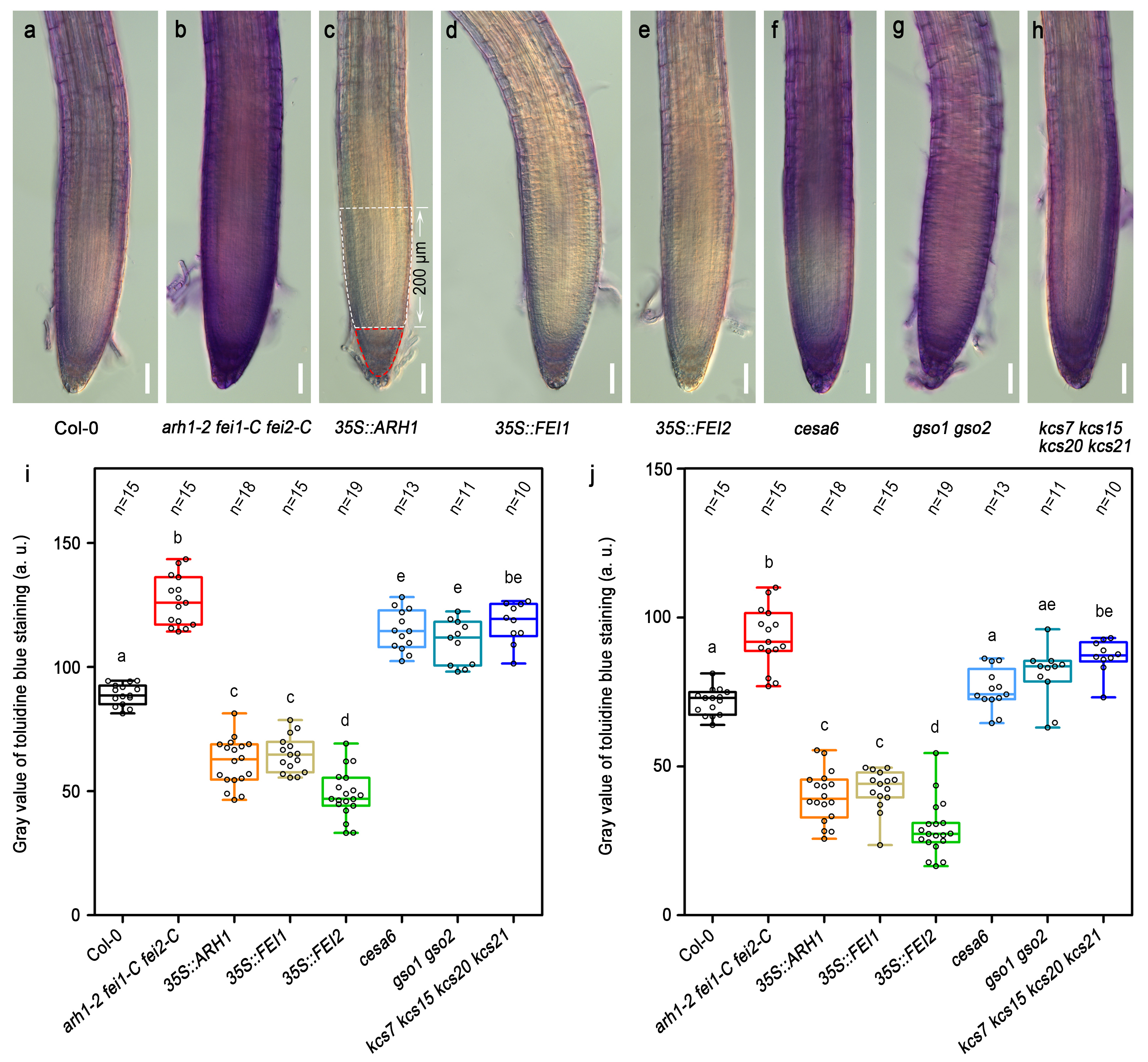


**Supplementary Figure 27 | Roots with defects in CCW show enhanced permeability to** **toluidine blue.**

**a-h**, Representative toluidine blue-stained four-day-old roots of Col-0 and other genotypes. **i**, Staining intensity for the root caps (red box as shown in c) of the roots represented in (a-h). **j**, Staining intensity for the meristematic region (200 µm starting from the quiescent center, white box as shown in c) of the roots represented in (a-h). Scale bars represent 50 µm. Boxplots span the first to the third quartiles of the data, and whiskers indicate the minimum and maximum values. The line in the box represents the mean. Each circle represents the measurement of an individual root. “n” represents the number of roots analyzed in the experiment. Three biological replicates were carried out. One-way ANOVA with Tukey’s multiple comparison test was used for statistical analyses with *P* < 0.01.


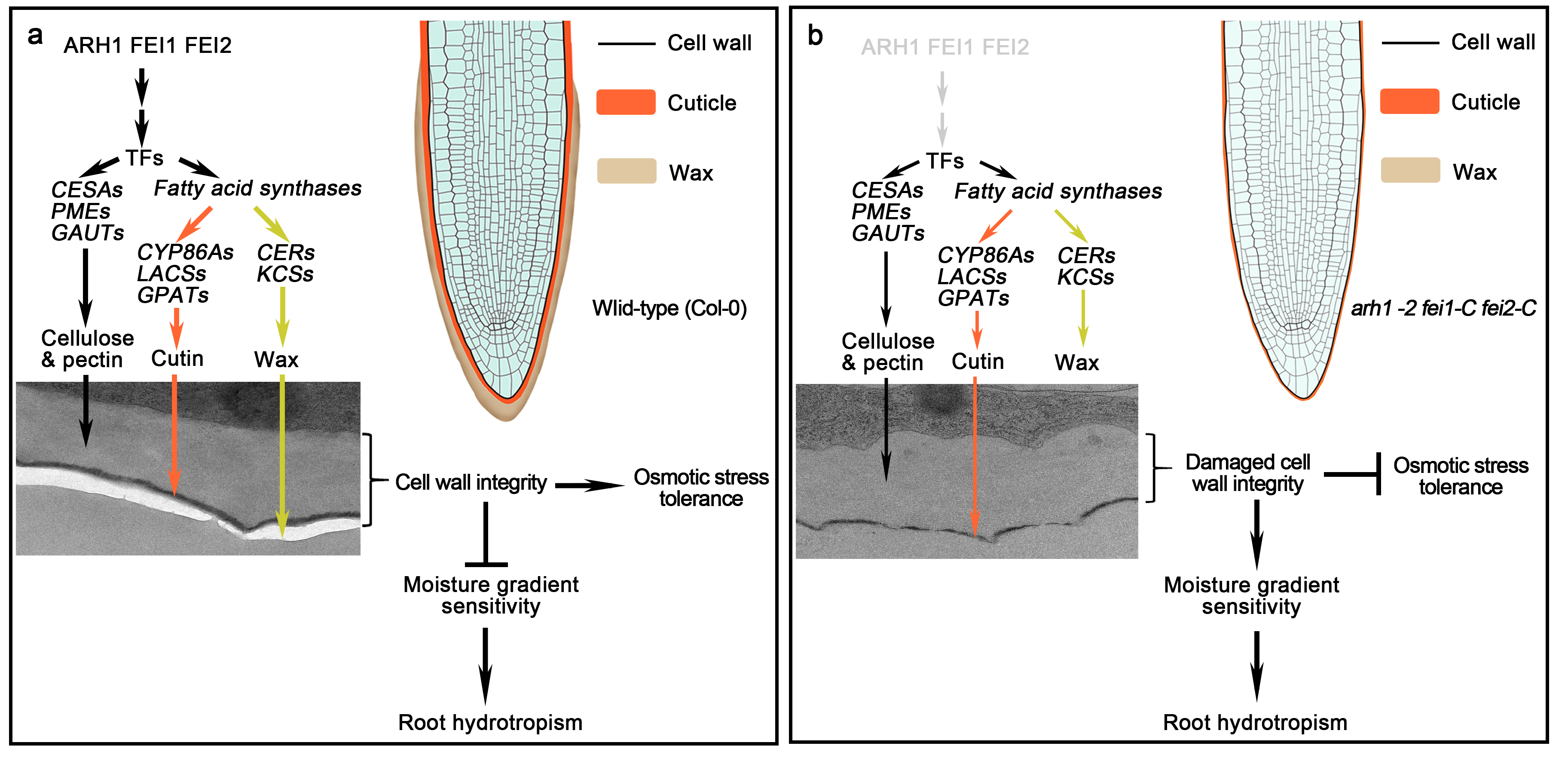


**Supplementary Figure 28 |** **A current model explaining how ARH1, FEI1, and FEI2 trade-off root hydrotropism and osmotic tolerance.**

**a**, Receptor-like kinases, ARH1, FEI1, and FEI2, can sense yet undefined extracellular signaling molecules and transduce them to downstream transcriptional factors (TFs), thereby activating the genes encoding for the biosynthetic enzymes of CCW. The intact cell wall and its depositions, such as cutin and wax, negatively regulate root tip response to moisture gradient, and positively regulate root tip tolerance to osmotic stress. **b**, Root tips of the *arh1-2* *fei1-C* *fei2-C* triple mutant have defects in CCW. They show enhanced sensitivity to moisture gradient and decreased tolerance to osmotic stress.

**Supplementary Table 1. Primers used for genotyping in this research.**

| Primer name | Sequence 5’ – 3’ |
| --- | --- |
| *arh1-1-F* | TCTCTCTCTCCTCTGCACCAG |
| *arh1-1-R* | GAAAATTCGCCCTCAAGTACC |
| *arh1-2-F* | TGATGATCGCTTTGGAGAATC |
| *arh1-2-R* | TAGCATTGGGAAGCTTAGTCG |
| *fei1-F* | CTTTTTCAATTCAGGTGCGTC |
| *fei1-R* | TTCAAGTAACTGGACAACCCG |
| *fei2-F* | TTGCCATCTATGGGAACTTTG |
| *fei2-R* | ATTACAACCATTGTTGCAGGC |
| *fei1- C-F* | TTCTTCCTCCTCTCGGATCCAT |
| *fei1-C-R* | ATGTGCATGAGAAGACATTATGCAG |
| *fei2- C-F* | TCTCACTGTTCTGGGTGCTG |
| *fei2- C-R* | TCACTCACAAGACGCATCAA |
| *cesa6-F* | GTTAGCGGCTTTCAAAGGATCA |
| *cesa6-R* | GGATGAGAAGTGAAGTCCATTTGA |
| *gso1-F* | TGCTCTCTCCAACCTTACTTCC |
| *gso1-R* | TGAAGGAGGTATCTCTCCTTCG |
| *gso2-F* | TCCCCATACTGTAAACATCGC |
| *gso2-R* | ATCGGTGACATGAAAAGCTTG |
| *kcs7-F* | TCTTTTCCCAGTTTGGTTTCT |
| *kcs7-R* | TCACTAATCCCTGATCGCTCT |
| *kcs15-F* | TGGCTTTCCCTTTGTCTACCT |
| *kcs15-R* | ATCACCATTGCTGCTTCTTCA |
| *kcs20-F* | CGCGGTTCTTGAGAAGACCGGTGTGA |
| *kcs20-R* | TAAAGACTACAAAGCCTGTCACTGTC |
| *kcs21-F* | TGCCGGTTTGCTAGTAGTGC |
| *kcs21-R* | TGCTTCTTGGGCTTAGTTTGTT |
| *bdg-F* | GAGAAAGCCAACAAGCTCATG |
| *bdg-R* | GTGTCACACACATAACGCGTC |
| *LBb1.3* | ATTTTGCCGATTTCGGAAC |
| *LB3* | TAGCATCTGAATTTCATAACCAATCTCGATACAC |

**Supplementary Table 2. Primers used for DNA or cDNA cloning in our research**

| Primer name | Sequence 5’ – 3’ |
| --- | --- |
| *pARH1-attB1- F* | AAAAAAGCAGGCTTCTTATTTTTAAGAGCTCGGCCCA |
| *ARH1-attB1- F* | GGGGACAAGTTTGTACAAAAAAGCAGGCTTCATGGGTATCTCGAATTGGGTTT |
| *ARH1-attB2-R* | AGAAAGCTGGGTCGAAGCCTAACGACATTAACATC |
| *pFEI1-attB1-F* | GGGGACAAGTTTGTACAAAAAAGCAGGCTTCATGGTGAAACAACGGACAACAA |
| *FEI1-attB1-F* | GGGGACAAGTTTGTACAAAAAAGCAGGCTTCATGATGGGCATCTGTGAGATGA |
| *FEI1- attB2-R* | GGGGACCACTTTGTACAAGAAAGCTGGGTCATCAGAGCTGGAATCATAAAATTCG |
| *pFEI2-attB1-F* | GGGGACAAGTTTGTACAAAAAAGCAGGCTTCGAAGAGTAGGGTGGAGCGTAATG |
| *FEI2-attB1-F* | GGGGACAAGTTTGTACAAAAAAGCAGGCTTCATGGGCATCTGTCTAATGAAGCG |
| *FEI2- attB2-R* | GGGGACCACTTTGTACAAGAAAGCTGGGTCATCGGAGCTGGAGTCGTAGAAG |

**Supplementary Table 3. Primers used for RT-qPCRs in our research**

| Primer name | Sequence 5’ – 3’ |
| --- | --- |
| *ARH1-qRT-F* | GGCGAATTTTCTCCAAGGCG |
| *ARH1-qRT-R* | AGCACCCTTGAGTGTATTGCT |
| *FEI1-qRT-F* | TCAAGCGATTAGTCCTGACGG |
| *FEI1-qRT-R* | TCTGGATCTTCCGGTCTCCA |
| *FEI2-qRT-F* | AAGGAGTGACCTGTGATGCAA |
| *FEI2-qRT-R* | CCAGCTTTCCAAGCTCAGGG |
| *CESA1-qRT-F* | CCCATGGCCATACGGTTTCT |
| *CESA1-qRT-R* | ACAGGTTGCCGTGGATCAAT |
| *CESA2-qRT-F* | TCGTCCCTGAGATAAGCAACTAC |
| *CESA2-qRT-R* | CCCCTCCGATTACCCAAAA |
| *CESA3-qRT-F* | CCCTATCACCTCCATTCCTCTTCT |
| *CESA3-qRT-R* | CGTCTATGCCTACGCCACTCC |
| *CESA4-qRT-F* | AGATGCGGAGTGGAAAGAACGTG |
| *CESA4-qRT-R* | GGTTGTCTTGCTTCAGCATCTAGG |
| *CESA5-qRT-F* | GATGCAATGGGGTAAAGTAGGG |
| *CESA5-qRT-R* | TGATGAGTAGTGTGGTTGGAGGG |
| *CESA6-qRT-F* | ACAGCACAGAAAGTGCCTGAG |
| *CESA6-qRT-R* | GGAGCATTTGATAGAACCCCA |
| *CESA7-qRT-F* | TAGATCGGCTTTCCCTCAGGTACG |
| *CESA7-qRT-R* | ACAGCACAGTGTTGGATGTGACG |
| *CESA8-qRT-F* | CCGGCAAATTTATCATCCCAACGC |
| *CESA8-qRT-R* | TTCGATACTGACTCCGCTCCATCG |
| *CESA9-qRT-F* | GGAGGGAGACTCATTGCTGG |
| *CESA9-qRT-R* | TGTATCGGGTTCCGCACTG |
| *CESA10-qRT-F* | ATTGGGGCAAAGAGATCGGG |
| *CESA10-qRT-R* | ACCCCTTGCATGCATCTTGA |
| *GAUT1-qRT-F* | CCGGGAAATTAGCAATGGCG |
| *GAUT1-qRT-R* | GCGCCATCCTCGGAATCTAA |
| *GAUT2-qRT-F* | TGCTTGTTGTTTGAAGGGAAAC |
| *GAUT2-qRT-R* | TCAGCTCATTAGCCCTTTCCA |
| *GAUT3-qRT-F* | CGTGACTGAAAAGCATATGGGC |
| *GAUT3-qRT-R* | TCACGGGGCAGTTTTACTCA |
| *GAUT4-qRT-F* | GTGAGAGAGCTTCGACTCCG |
| *GAUT4-qRT-R* | CTTTGGCCAGTGTTTGCTCC |
| *GAUT5-qRT-F* | ACATCTGTGGAAAGCAGGGG |
| *GAUT5-qRT-R* | GCTTGCCCTGAGTCCTGATT |
| *GAUT6-qRT-F* | TCGTCGATGGCAGAGGATTT |
| *GAUT6-qRT-R* | AGACGAAACTGACCAACGGG |
| *GAUT7-qRT-F* | GAGAAGGCATCTGAGTCGGG |
| *GAUT7-qRT-R* | AAAAGCATTGGCCGTGTAGC |
| *GAUT8-qRT-F* | ATAGTTTGGCCGCGTACGAT |
| *GAUT8-qRT-R* | CCATTGGGGAGAGAGCACTT |
| *GAUT9-qRT-F* | GCCAATCTACCGGACTTCGT |
| *GAUT9-qRT-R* | AGGCTGATGACGAGAGGAGT |
| *GAUT10-qRT-F* | CACAGCTCTGCGAGTACAGT |
| *GAUT10-qRT-R* | GCAGGAGGGCAATACCAGAG |
| *GAUT11-qRT-F* | CGTGACTGCTCGGTACCATT |
| *GAUT11-qRT-R* | CTGTCCAGTGGCTCTGTGAG |
| *GAUT12-qRT-F* | GGCATATGCTTGGTCTCGGA |
| *GAUT12-qRT-R* | GCCAAGGCTTAGCTCTTCCA |
| *GAUT13-qRT-F* | CGATTGGGCATGAAAGTCGG |
| *GAUT13-qRT-R* | AAGTGATTTCGCCGGTGGAT |
| *GAUT14-qRT-F* | TGCGGGTATGCATTCATGGT |
| *GAUT14-qRT-R* | TGGCTTTCCACAGCTTCCAA |
| *GAUT15-qRT-F* | CCCTGAGGTACGATCTCTTTGG |
| *GAUT15-qRT-R* | GCCGCAAGTATTGGCTTCAT |
| *QUA2-qRT-F* | ACCAAAGCCAACGGAAGTGT |
| *QUA2-qRT-R* | TCCAATCTCGACACAGCTCG |
| *QUA3-qRT-F* | ACTGTCACTTGCTCTCCAGC |
| *QUA3-qRT-R* | ACAACCACGTGTGACCAAGA |
| *CGR2-qRT-F* | AAGCATGGGGTGTTGAACCA |
| *CGR2-qRT-R* | AAGGATTTTGCCCGGTAGGG |
| *CGR3-qRT-F* | AGGCAAGTAAGGCGTGTAGG |
| *CGR3-qRT-R* | ATGCTCCCACGAGAACAAGG |
| *PME2-qRT-F* | CCGCAAGCTCAAGGAGGTTA |
| *PME2-qRT-R* | AAAGTCACCGCTTCCGTCAT |
| *PME5-qRT-F* | CGGGTTTTACGGTGCACAAG |
| *PME5-qRT-R* | ATGGAGCGGCCATTACCAAA |
| *PME8-qRT-F* | CCACGGTCCAAGTCTTTGGT |
| *PME8-qRT-R* | TCTTATTGCCACCGCCTGAG |
| *PME14-qRT-F* | GGCACGTACCAAAACCACAC |
| *PME14-qRT-R* | TTGGGTCGGACTTGATGGTG |
| *PME41-qRT-F* | GCTAACGCAATCGCGGAAAT |
| *PME41-qRT-R* | TAACGGCAGTCTTGGAGAGC |
| *PME44-qRT-F* | GAACGCAAATGTTGGGTGGG |
| *PME44-qRT-R* | CCAACCACCTCTCTGTCCAC |
| *PME46-qRT-F* | GTTGGAGACTGGTAACGCCA |
| *PME46-qRT-R* | GTCCGATATTTCCCCGACCC |
| *PME53-qRT-F* | GAATGGGGAGACACAGCACA |
| *PME53-qRT-R* | TGGGGTCGTGTTCCTGAATG |
| *ACC1-qRT-F* | TTTAGTGGGTTTGGGGAGTTT |
| *ACC1-qRT-R* | TTAACCGAGCCAGCCATTGT |
| *ACC2-qRT-F* | TGGTGTTTTGGAGTTGCTTGT |
| *ACC2-qRT-R* | TTTGCAACATTGGGCTGTCG |
| *KASI-qRT-F* | GAGGTCTAGAAGCCATCGCC |
| *KASI-qRT-R* | CTGGGTTTGAAAGCGGAATCA |
| *KASII-qRT-F* | GGCTGAGATGGATCATTGGCA |
| *KASII-qRT-R* | AGAGCATTTCTGGACCGACAA |
| *KASIII-qRT-F* | CCATCCTCTCTCACACACACC |
| *KASIII-qRT-R* | CGGATACAACGAATCGCAAGG |
| *FTAT1-qRT-F* | TCACTGGTGAAGTCACTGGC |
| *FATA1-qRT-R* | AGACCAAGTACTCGTCCCGA |
| *FATA2-qRT-F* | GGGTCCTTGAGAGCATACCG |
| *FATA2-qRT-R* | GGGTCCTTGAGAGCATACCG |
| *FATB-qRT-F* | GCGATATCGGTAACCTGGCA |
| *FATB-qRT-R* | TTCTTACGGTGCAGTTCCCC |
| *CER1-qRT-F* | GTTCAGGTCTCCACTCTGCG |
| *CER1-qRT-R* | CCAGCCATACCTTCACCCAA |
| *CER2-qRT-F* | TGCTTGTTGTTGGATGGATCTG |
| *CER2-qRT-R* | ATGTGGGTGGGATTTGGGTC |
| *CER3-qRT-F* | AGAGAGTGCCGGAGTTTGTG |
| *CER3-qRT-R* | AATGGCCACATCGGTAGCAA |
| *CER4-qRT-F* | CGTACCGTATGGGTGAGACG |
| *CER4-qRT-R* | CCGATTACTCGGAGCTGGTC |
| *CER6-qRT-F* | TTGGGTTGACCATCCATCCAG |
| *CER6-qRT-R* | ATGACTGTCCATGCACCACA |
| *CER8-qRT-F* | CCAACATCCATTTCTCAGCTCC |
| *CER8-qRT-R* | TGGTTTGAATCCGGTAGGTCG |
| *MAH1-qRT-F* | AGTATTAGAGCATCCTCGTCCA |
| *MAH1-qRT-R* | GACCGGGCAGACACATTTTG |
| *KCS1-qRT-F* | GGTCTAACCAGGCGGTTCAA |
| *KCS1-qRT-R* | GACCGGTTAGCCACGTAGAG |
| *KCS2-qRT-F* | GGTCTCAGCGTGTCGGTATC |
| *KCS2-qRT-R* | TAGGCGGGACACGTAAGAGA |
| *KCS3-qRT-F* | ACCCCAAAAATTTTAATGTTACGCT |
| *KCS3-qRT-R* | TTGAACAACAACTAAAAGTGGCA |
| *KCS4-qRT-F* | ATCCGACAAACACGGATGCT |
| *KCS4-qRT-R* | CGGAGATAACAACGGCGAGA |
| *KCS5-qRT-F* | GGGTTTGGCAGATCGCTTTC |
| *KCS5-qRT-R* | ATACAATCCGACCACGCTCC |
| *KCS7-qRT-F* | TTGAAGGCGTGGAGAAGCAT |
| *KCS7-qRT-R* | TGCAGCTCATACCACAACGA |
| *KCS8-qRT-F* | ACCCTAATGGGACACGCAAG |
| *KCS8-qRT-R* | TCTTGACCAAGACCGGAACG |
| *KCS9-qRT-F* | AACTCGAAGCTGAAGCCGTA |
| *KCS9-qRT-R* | CATGAGTCTGCGAAAGCTGC |
| *KCS10-qRT-F* | CGTTCTCGGTTAGGGTCAGG |
| *KCS10-qRT-R* | AACCGGTATGGTCGCCAAAT |
| *KCS11-qRT-F* | TTCTACAACGTTCGGGGCTC |
| *KCS11-qRT-R* | ACAGTCTCGGCCTCTTTTCG |
| *KCS12-qRT-F* | TCAAGGTCCCTGACGCTTTC |
| *KCS12-qRT-R* | AGGTTTTCCGATTGCTAAACACG |
| *KCS13-qRT-F* | TCATTGGTGGGTAACCCGTG |
| *KCS13-qRT-R* | ACTTATAACGTGACTTCACACGC |
| *KCS14-qRT-F* | TTGCAATGGCAGATTTCAAGC |
| *KCS14-qRT-R* | CGATCTTGACCGGAAAAGGAGA |
| *KCS15-qRT-F* | CCCGATCATCTCAAGATCACA |
| *KCS15-qRT-R* | CCCTGATTGGTCCAGCACTT |
| *KCS16-qRT-F* | CTTTACCTCGCTCTCGGGTC |
| *KCS16-qRT-R* | CATGATCCTCTGGGTGCTGG |
| *KCS17-qRT-F* | GCTCTGGTCTCGGTGAAGAG |
| *KCS17-qRT-R* | CGAAGATTACCTGCTCCGCT |
| *KCS18-qRT-F* | ATCCGTCCTCGCTCGATTTC |
| *KCS18-qRT-R* | TTCACGTGACGCTGCAAAAG |
| *KCS19-qRT-F* | CCGAGACAATGGGTCCTCAC |
| *KCS19-qRT-R* | GACCACTGTCACGAGCTTCA |
| *KCS20-qRT-F* | CCAACACCGTCACTTTCTGC |
| *KCS20-qRT-R* | GGCCAGCACTACATCCCATT |
| *KCS21-qRT-F* | AACTTCACTGGGACCTCTGG |
| *KCS21-qRT-R* | GCAGAAATGCTCAAACGCTG |
| *CYP86A1-qRT-F* | ACTTCTTCGGTGGCCTTGAG |
| *CYP86A1-qRT-R* | GAACTCTAACGGCTCCTCCG |
| *CYP86A2-qRT-F* | GGGTACACTCTCTCAACAATCCA |
| *CYP86A2-qRT-R* | GGCATTAAGAAGTGGAGGCG |
| *CYP86A4-qRT-F* | TGCCATGCTTCTTGTAGCGA |
| *CYP86A4-qRT-R* | ACTACCCAACAGAGGCCAGA |
| *CYP86A7-qRT-F* | TCAACGTAAGACCGCTGCAT |
| *CYP86A7-qRT-R* | AGACGGTTCTTGATGGCTCG |
| *CYP86A8-qRT-F* | TTAACTCCGACGGTGACACG |
| *CYP86A8-qRT-R* | CCGAGCCGAGCATTTTCAAG |
| *LACS1-qRT-F* | CGGTCGCGACTTTTGTTGTT |
| *LACS1-qRT-R* | GAAGCGCCTTTGCGAAAGAA |
| *LACS2-qRT-F* | TGGAGAATGGCAAGAAGATGGA |
| *LACS2-qRT-R* | TCTGAGCAATGAGGGGACATC |
| *LACS3-qRT-F* | GGAAAGCAAGGCGTTGATGG |
| *LACS3-qRT-R* | ACGAGATCATCAGGCGGTTC |
| *LACS4-qRT-F* | GATTTCCCGACCCGATCGAA |
| *LACS4-qRT-R* | TCCATCTACAATCTCGCGGC |
| *LACS5-qRT-F* | TGGTATCCAAAGCTGCTGGG |
| *LACS5-qRT-R* | TCCACACGTACTTTCCTGCC |
| *LACS6-qRT-F* | CATGCAACTGTGCAAGCCAT |
| *LACS6-qRT-R* | TGATGAGGGAAGCGAGGGTA |
| *LACS7-qRT-F* | GGTTCCAGTGTGGAAGCAGA |
| *LACS7-qRT-R* | GAAACCGACAGCAACACCAC |
| *LACS8-qRT-F* | TGTGCTTGGAGGACACATCC |
| *LACS8-qRT-R* | TGGGAGACCCCATGCAGATA |
| *LACS9-qRT-F* | CGAGTGGCTCACTTTTGGGA |
| *LACS9-qRT-R* | TGAAGCAACCCTGTAGGGAG |
| *GAPT1-qRT-F* | CGACCCAGTTTTCCTCACCA |
| *GAPT1-qRT-R* | AGTCTCTGCATTGCCTCACC |
| *GAPT2-qRT-F* | GAAGCCGGAGGGGTGATAAG |
| *GAPT2-qRT-R* | TGATTTCCCCACTCGGAAGC |
| *GAPT3-qRT-F* | CCCATCAATTGCTCTCCAACA |
| *GAPT3-qRT-R* | CTTGGCGGACATGTTTGGATT |
| *GAPT4-qRT-F* | ACATCGAACTCGTCTCTCGC |
| *GAPT4-qRT-R* | CGGATTCGCAGTCACCACTA |
| *GAPT5-qRT-F* | AACCACGGTGACCAACAACT |
| *GAPT5-qRT-R* | GGAGGATGATGAGAGCGGTG |
| *GAPT6-qRT-F* | CAAGACGGACCACGACTTCA |
| *GAPT6-qRT-R* | GCGTTGGACTAATCTGCCCT |
| *GAPT7-qRT-F* | GGTCATAACCAGCGGCTACA |
| *GAPT7-qRT-R* | AAGGATGATGAGTGCGGTGG |
| *GAPT8-qRT-F* | TGCAGAGACGGAGAGTACGA |
| *GAPT8-qRT-R* | CAACAGCGACGAGCATGAAG |
| *GAPT9-qRT-F* | CTATGAGCAGTACGGCAGGG |
| *GAPT9-qRT-R* | TCTAGCAAATCACGCAGGCT |
| *EXPA1-qRT-F* | ACAACAAGCGATGGCCAAAC |
| *EXPA1-qRT-R* | ACTGCTTCTACTGTGAAGGTCTG |
| *EXPA2-qRT-F* | CACAATGGGTGGTGCTTGTG |
| *EXPA2-qRT-R* | AAAGCAGGCCCCACATTTCT |
| *EXPA12-qRT-F* | TGTGGGGATGTGTTCTAACGG |
| *EXPA12-qRT-R* | ATGGCCTTTTTACCAAGTGAAG |
| *EXPA15-qRT-F* | ATCAAGTGTCAGAGCGACGG |
| *EXPA15-qRT-R* | GAAGCGGAGGGTTACACCAA |
| *EXPA17-qRT-F* | CATGCGACCTTTTATGGCGG |
| *EXPA17-qRT-R* | CGCGGTGTTTGTCTTGTAGC |
| *EXPA18-qRT-F* | CGCCTAACTGCTACTACGGG |
| *EXPA18-qRT-R* | AAATGGACTCGTGGCGGATT |
| *ACT2-qRT-F* | TTGTTTGTTTCATTTCCCTTTTTG |
| *ACT2-qRT-R* | GCAGACGTAAGTAAAAACCCAGAGA |
