# Supplementary material for: Defects in the cell wall and its deposition caused by loss-of-function of three RLKs alter root hydrotropism in Arabidopsis thaliana
Source: Nat Commun. 2024 Mar 26;15:2648. doi: 10.1038/s41467-024-46889-2 (PMC10966064; doi:10.1038/s41467-024-46889-2)
Supplement: Supplementary file 3 — Description of Additional Supplementary Files [file 41467_2024_46889_MOESM3_ESM.pdf]

Description of Additional Supplementary Files:

Supplementary Movie 1: The triple mutant arh1-2 fei1-C fei2-C shows an enhanced root hydrotropic response. Four-day-old seedlings of wild-type (Col-0) and arh1-2 fei1-C fei2-C were transferred from 1/2 MS medium to a hydrostimulation medium (1/2 MS medium supplemented with 400 mM D-sorbitol at the bottom-right side). The movie was generated using images obtained from a continuous 16-hour imaging session, which started immediately following the initiation of hydrostimulation.

Supplementary Movie 2: Roots of the triple mutant show a gravitropic response similar to those of Col-0. Four-day-old seedlings of wild-type (Col-0) and arh1-2 fei1-C fei2-C were horizontally placed to observe root gravitropism. The movie was generated using images obtained from a continuous 19-hour imaging session, starting immediately after seedlings been horizontally placed.
